# Regularly Arranged Micropore Architecture Enables Efficient Lithium-Ion Transport in SiO_*x*_/Artificial Graphite Composite Electrode

**DOI:** 10.1007/s40820-025-01929-4

**Published:** 2025-10-09

**Authors:** Jaejin Lim, Dongyoon Kang, Cheol Bak, Seungyeop Choi, Mingyu Lee, Hongkyung Lee, Yong Min Lee

**Affiliations:** 1 Department of Chemical and Biomolecular Engineering, Yonsei University, 50 Yonsei-ro, Seodaemun-gu, Seoul, 03722 Republic of Korea; 2 Department of Energy Science and Engineering, Daegu Gyeongbuk Institute of Science and Technology (DGIST), Daegu, 42988 Republic of Korea; 3 Department of Battery Engineering, Yonsei University, 50 Yonsei-ro, Seodaemun-gu, Seoul, 03722 Republic of Korea; 4 Department of Materials Science and Engineering, Yonsei University, 50 Yonsei-ro, Seodaemun-gu, Seoul, 03722 Republic of Korea

**Keywords:** Lithium-ion battery, SiO_*x*_/artificial graphite composite electrode, Microstructure, Pore, Perforated current collector

## Abstract

To enhance the electrochemical performance of lithium-ion battery anodes with higher silicon content, it is essential to engineer their microstructure for better lithium-ion transport and mitigated volume change as well. Herein, we suggest an effective approach to control the micropore structure of silicon oxide (SiO_*x*_)/artificial graphite (AG) composite electrodes using a perforated current collector. The electrode features a unique pore structure, where alternating high-porosity domains and low-porosity domains markedly reduce overall electrode resistance, leading to a 20% improvement in rate capability at a 5C-rate discharge condition. Using microstructure-resolved modeling and simulations, we demonstrate that the patterned micropore structure enhances lithium-ion transport, mitigating the electrolyte concentration gradient of lithium-ion. Additionally, perforating current collector with a chemical etching process increases the number of hydrogen bonding sites and enlarges the interface with the SiO_*x*_/AG composite electrode, significantly improving adhesion strength. This, in turn, suppresses mechanical degradation and leads to a 50% higher capacity retention. Thus, regularly arranged micropore structure enabled by the perforated current collector successfully improves both rate capability and cycle life in SiO_*x*_/AG composite electrodes, providing valuable insights into electrode engineering.

**Supplementary Information:**

The online version contains supplementary material available at 10.1007/s40820-025-01929-4.

## Highlights

- The internal pores of the electrode were engineered into a regularly arranged micropore (RAM) structure by introducing a perforated and surface-modified Cu current collector (pCu).
- The pore network, favorable for fast ion transport, effectively mitigates concentration polarization and enables uniform ion distribution, contributing to high-rate operation of lithium-ion batteries.
- The RAM structure, featuring a unique interlocking electrode configuration and hydroxyl-rich pCu surface, suppressed mechanical degradation and improved long-term cyclability by 50%.

**Supplementary Information:**

The online version contains supplementary material available at 10.1007/s40820-025-01929-4.

## Graphical abstract

**Figure:**
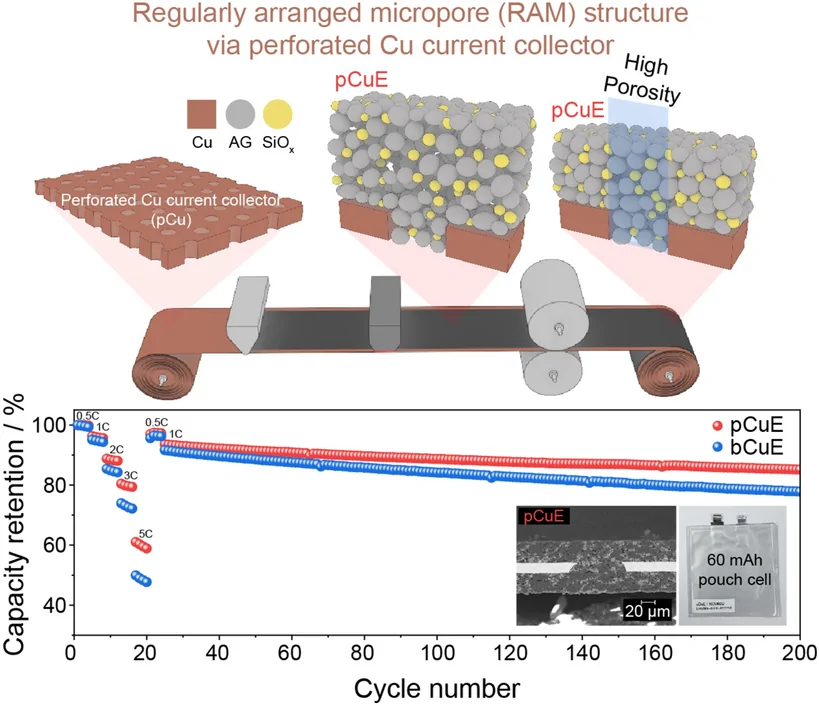

## Introduction

Lithium-ion batteries (LIBs) play a pivotal role in the electrified world due to their high energy density, long life, and cost-effectiveness [1–4]. The electrification of transportation with electric vehicles (EVs) represents a major trend in this global shift [5]. To facilitate the successful adoption of EVs, current LIBs face several goals—high power performance, high safety, high energy density, and low cost, needing cutting-edge electrode design [6]. Of these, power delivery is particularly critical, as large-scale batteries in EVs are required to provide substantial power output, often in the tens of kilowatts. Therefore, high discharging power supports the operation of industrial, large, and heavy-duty vehicles [7, 8], while high charging power is essential for the rapid charging of EVs.

Various studies at the electrode level have aimed to improve the power and rate capabilities of batteries. One strategy involves the use of silicon-based materials in anodes [9–11]. Silicon offers a gravimetric capacity up to 3579 mAh g^−1^ (at room temperature for Li_15_Si_4_), significantly higher than the theoretical gravimetric capacity of traditional graphite anodes, which is 372 mAh g^−1^ [12, 13]. Consequently, silicon-based anodes can achieve thinner electrodes for the same areal capacity compared to graphite-based materials, since thick electrodes impede lithium-ion diffusion within the electrode, which is a limiting factor in achieving sufficient rate capability [14–16]. An anode that incorporates silicon materials can overcome the limitations posed by thick electrodes, thereby enhancing the high-power capability of the battery [17, 18]. Nonetheless, the application of such anode designs is generally challenging due to the substantial volume expansion (> 300%) associated with lithiation in silicon-based materials [19–21]. As an alternative, silicon oxide (SiO_*x*_, *x* < 2) can be utilized, where Si nanoparticles are embedded within an SiO_2_ matrix [22–29]. The formation of Li_4_SiO_4_ and Li_2_O during lithiation acts as a buffer domain, accommodating volume expansion with a relatively lower expansion rate (ca. 200%) [30, 31]. However, like pure Si, the mechanical degradation and insulating nature of SiO_*x*_ still can lead to large irreversible capacities during cycling [32–35].

Another approach to enhancing rate capabilities involves tailoring diffusion pathways. Concentration polarization, a result of lithium-ion diffusion constraints within the electrolyte, critically impedes high-rate battery operations [36–41]. By strategically modifying lithium-ion diffusion pathways within the electrode, one can effectively prevent such polarization. For instance, Chen et al. demonstrated that creating vertical channels through the electrode thickness via laser patterning facilitates lithium transport into the bulk electrode under fast charging conditions surpassing a 4C-rate [42]. Similarly, Li et al. developed a low-tortuosity electrode by embedding ferromagnetic Fe_3_O_4_ nanoparticles in an oil emulsion within the electrode slurry and subsequently aligning this emulsion using a magnetic field after slurry coating [38, 43]. Dang et al. employed vacuum drying after slurry casting to direct pore orientation toward the electrode's top, decreasing pore tortuosity [44]. This precise control over pore structures enhances lithium-ion transport and supports high-capacity retention during rapid charge/discharge cycles. Nonetheless, existing studies reveal that manipulating internal pore structure typically requires additional processing steps or additives.

Herein, we enhance the power performance of the SiO_*x*_/artificial graphite (AG) composite electrode through a regularly arranged micropore (RAM) architecture. The RAM features alternating high and low porosity in the electrode's in-plane direction, and it was achieved without additional processes, simply by incorporating a perforated Cu current collector (pCu) with micrometer-scale patterned perforation. Cross-sectional scanning electron microscopy (SEM) analysis reveals that casting a slurry onto the pCu and subsequent calendaring resulted in lower porosity in the non-perforated regions (NPRs) and higher porosity in the perforated regions (PRs), forming the unique RAM structure. Rate tests on the SiO_*x*_/AG||LiNi_0.6_Co_0.2_Mn_0.2_O_2_ full cell confirm that the RAM structure supports increased capacities at high rates and effectively mitigates lithium-ion concentration polarization in the electrolyte, as evidenced by diffusion simulations. Moreover, 0.5C-rate cycle tests demonstrate that the pCu prevents delamination between the electrode and current collector and effectively reduces mechanical degradation of the composite electrode. We expect that the integrating the pCu with silicon-based electrodes and engineering the pore microstructure as bridging technologies will enhance the power characteristics of LIBs.

## Experimental Section

### Current Collector Characterization

The surface morphology of the perforated Cu (pCu) and the bare Cu (bCu) current collectors was examined using field-emission scanning electron microscopy (FE-SEM, S-8020, Hitachi, Japan) at an acceleration voltage of 3.0 kV. The contact angles of the pCu and the bCu with deionized water were measured using a drop shape analyzer (DSA 100, KRUSS, Germany) to assess the hydrophilicity of the Cu current collectors. Surface functional groups on the pCu and the bCu were identified using X-ray photoelectron spectroscopy (XPS, ES-CALAB 250Xi, Thermo Scientific, USA). Magnetic field imaging (MFI) measurements of the pCu and bCu were conducted using a B-Lab 160S system (DENKweit, Germany), equipped with a movable magnetic sensor comprising a 64-magnetometer array (16 cm) spaced at 2.5 mm intervals. For the measurement, Cu foils were cut into 10 × 50 mm^2^, and a current of 3 A was applied to assess the magnetic field distribution. The mechanical properties of the pCu and bCu were evaluated using a universal testing machine (UTM, United Calibration, USA) with a tensile speed of 10 mm min^−1^ and specimen dimensions of 30 × 100 mm^2^.

### Electrode Fabrication

To fabricate electrodes with the perforated (pCu) and the bare Cu (bCu) current collectors, a slurry was prepared by planetary mixing silicon oxide (SiO_*x*_, *x* < 2, Osaka Titanium Technology, Japan), artificial graphite (AG, SCMG-AR, Showa Denko, Japan), Super P (Imerys, Switzerland), polyacrylic acid (PAA, Sigma-Aldrich, USA) binder in a weight ratio of 31:62:3:4.0 with deionized water as the solvent. The slurry was cast onto the pCu and the bCu foils (10 µm, Lotte Energy Materials, Republic of Korea) using a doctor blade and dried at 60 °C for 2 h. After drying, the anodes were calendered to achieve a target density of 1.5 g cm^−3^. The mass loading for all anodes was controlled at 4.95 mg cm^−2^ to ensure a consistent areal capacity of 3.6 mAh cm^−2^.

### Cell Assembly

A 2032 coin-type half-cell was assembled with a lithium metal foil (200 µm, Honjo Metal, Japan), a polyethylene (PE) separator (F20BHE, 20 µm, Tonen, Japan), and the as-prepared SiO_*x*_/AG electrode in an Ar-filled glove box. Before assembly, the lithium metal foil, PE separator, and SiO_*x*_/AG electrode were punched to diameters of 16.2, 18, and 12 mm, respectively, and the punched SiO_*x*_/AG electrode was dried in a vacuum oven at 60 °C for 12 h. The electrolyte used for all 2032 coin-type half-cells was 1.15 M lithium hexafluorophosphate (LiPF_6_) in ethylene carbonate (EC)/ethyl methyl carbonate (EMC) (3:7 v/v) with 2 wt% fluoroethylene carbonate (FEC) additive (Enchem, Republic of Korea).

For the full cells, a cathode slurry was prepared using LiNi_0.6_Co_0.2_Mn_0.2_O_2_ (NCM622, L&F, Republic of Korea), Super P (Imerys, Switzerland), and polyvinylidene fluoride (PVDF, KF1300, Kureha, Japan) in a weight ratio of 96:2:2 with N-methyl-2-pyrrolidone (NMP, Sigma-Aldrich, USA) as the solvent. The slurry was cast onto aluminum (Al) foils (15 µm, Sam-A, Republic of Korea) and dried at 120 °C for 2 h. The cathodes were then calendered to achieve a target density of 3.5 g cm^−3^. The mass loading and areal capacity of the cathode were 19.8 mg cm^−2^ and 3.3 mAh cm^−2^, respectively, with an N/P ratio of ~ 1.1. The prepared anodes and cathodes were punched to diameters of 16 and 14 mm, respectively, and dried in a vacuum oven at 60 °C for 12 h. 2032 coin-type SiO_*x*_/AG||NCM622 full cells were assembled in an Ar-filled glove box with an 18-mm-diameter PE separator (F20BHE, 20 µm, Tonen, Japan) and the same liquid electrolyte as used in the half-cells.

Single-layer pouch-type full cells were assembled in a dry room with a dew point below -60 °C. The as-prepared SiO_*x*_/AG anode and NCM622 cathode were punched to dimensions of 25 × 25 and 23 × 23 mm^2^, respectively. A PE separator (14 µm, W-Scope Korea, Republic of Korea) was used for all pouch cells, which punched to dimension of 30 × 30 mm^2^. All electrodes and separators were dried in a vacuum oven at 60 °C for 12 h before assembly. After welding the lead tab to the electrodes, each layer of anode, separator, and cathode was stacked in an Al pouch film (thickness = 150 µm, DNP, Japan). The pouch cells were sealed under vacuum using degassing machine after injecting the liquid electrolyte consisting of 1.15 M LiPF_6_ in EC/EMC (3:7 v/v) with 2 wt% FEC additive (Enchem, Republic of Korea).

Three-electrode pouch-type full cells were assembled following the same procedure aforementioned, with lithium metal foil (200 µm, Honjo Metal, Japan), SiO_*x*_/AG, and NCM622 electrodes serving as the reference, counter, and working electrodes, respectively.

Two-stack pouch cells with a nominal capacity of 60 mAh were also fabricated using double-sided pCuE and bCuE electrodes. The fabrication process was identical to that described above, except for the dimensions of the electrodes and separator. Specifically, the SiO_*x*_/AG anode, NCM622 cathode, and separator were punched into dimensions of 35 × 35, 33 × 33, and 40 × 40 mm^2^, respectively.

### Electrochemical Measurement

All 2032 coin-type half-cells were aged for 12 h before electrochemical measurements. To stabilize the cells, a two-step precycling procedure was conducted, including a formation cycle and three stabilization cycles. During the formation cycle, the cell was charged (lithiated) at a 0.1C-rate in constant current (CC) mode to a cutoff voltage of 0.005 V and discharged (delithiated) at the same C-rate to a 1.5 V cutoff voltage. The cell was then cycled three times at a 0.2C with a CC/constant voltage (CV) charge to 0.005 V and a cutoff current of 0.02C, followed by a 0.2C discharge to 1.5 V. All cycles were conducted at 25 °C using a battery cycler (WBCS3000L, Wonatech, Republic of Korea).

Direct current internal resistance (DCIR) of the half-cell was measured using a hybrid pulse power characterization (HPPC) protocol, comprising a 5C discharge for 10 s, followed by a 40 s rest, a 3.75C discharge for 10 s, another 40 s rest, and finally a 1.25C discharge for 10 s [45]. DCIR measurements were repeated every 10% between states of charge (SoC) from 10 to 100%. Electrochemical impedance spectra (EIS) were collected using a potentiostat (VMP-300, BioLogic, France) with a 10 mV amplitude and frequencies ranging from 5 MHz to 50 mHz.

For 2032 coin-type full cells and pouch-type full cells, aging was conducted for 12 and 24 h respectively. The same two-step precycling procedure was applied. During the formation cycle, the cells were charged at 0.1C in CC mode to 4.3 V and discharged at the same rate to 3.0 V. Subsequently, the cells were cycled three times at 0.2C with a CC/CV charge to 4.3 V and a cutoff current of 0.02C, followed by a 0.2C discharge to 3.0 V. For rate testing, the discharge rate was varied from 0.2 to 5C (0.2C, 0.5C, 1C, 2C, 3C, 5C, and back to 0.2C) in CC mode, while the charge rate was maintained at 0.2C in CC/CV mode. For cycle testing, a 0.5C CC/CV charge and a 0.5C CC discharge mode were used. The voltage range was set between 3.0 and 4.3 V for both rate capability and cycle tests. All cycles were conducted at 25 °C using a battery cycler (WBCS3000L, Wonatech, Republic of Korea). EIS measurements for full cells were conducted under the same conditions as those for the half-cells.

### Electrochemical Dilatometry

Operando electrochemical dilatometry was performed to evaluate the thickness changes of the pCu-adopted electrode (pCuE) and the bCu-adopted electrode (bCuE) during charging and discharging using a surface and interfacial cutting analysis system (SAICAS, SAICAS-DN, Daipla Wintes, Japan). A 2-mm-diameter cylindrical tip was placed on the surface of the prepared three-electrode pouch-type full cell. Thickness changes were measured with a displacement sensor with 100 nm resolution, maintaining a cell pressure of 1 MPa. Electrochemical tests were carried out using a VMP-300 potentiostat, with a single charge and discharge procedure. The cell was charged at 0.2C in CC mode to 4.3 V with a time limit of 7,200 s and discharged at 0.2C in CC mode to 3.0 V with the same time limit.

### Electrode Characterization

Confocal microscopy (VHX-900F, Keyence, Japan) was utilized to obtain optical images of SiO_*x*_/AG electrode. To further analyze the microstructure of the electrodes before and after cycling, cross-sectional SEM images were acquired using an ion milling system (ArBlade 5000, Hitachi, Japan) and FE-SEM with energy-dispersive X-ray analysis (FE-SEM/EDX, S-8020, Hitachi, Japan). An electrode resistance measurement system (RM2610, HIOKI, Japan) was used to measure the interfacial electronic resistivity between the current collector and composite electrode, as well as the bulk electronic resistivity of the composite electrode. Measurements were conducted in the current range of 0–1 mA and voltage range of 0–0.5 V. The adhesion strength of the electrode was assessed using a surface and interfacial cutting analysis system (SAICAS, SAICAS-DN, Daipla Wintes, Japan). Adhesion strength within the electrode was measured five times at the interface between current collector and composite electrode using a boron nitride blade (width: 1 mm) at a horizontal speed of 2 µm sec^−1^. X-ray computed tomography (XCT, ZEISS Xradia 510 Versa, Germany) was employed to non-destructively evaluate the internal structure of the pCuE-DS over a wide electrode area (500 × 500 μm^2^) with a voxel size of 700 nm. The imaging was performed at an accelerating voltage of 140 kV, with one projection acquired every 3 s, resulting in a total of 3,201 projections.

### Pore Structure Characterization

The pore structure of the pCuE and the bCuE was analyzed based on cross-sectional SEM images and a two-dimensional (2D) pore network model (PNM). SEM images were segmented using GeoDict2023 (Math2Market, Germany) and MATLAB (MathWorks, USA) with gray value thresholding and marker-based watershed segmentation algorithms [46]. Based on segmented 2D label images, each pore was identified, and porosity distribution, equivalent pore radius, and coordination number were calculated using built-in MATLAB functions and open-source PNM analysis code.

### Electrochemical Modeling and Simulation

An electrochemical model for the SiO_*x*_/AG||NMC622 full cell was developed using COMSOL Multiphysics 6.1 (COMSOL Inc. USA), based on Doyle and Newman's approach [47]. The model was constructed in a pseudo-four-dimensional (P4D) space with Cartesian dimensions x, y, z, and a pseudo-dimension r. For simulating the CC discharging process at 3C, boundary conditions of 4.3 V and 3C were defined. Overpotential, depth of discharge (DoD), and lithium-ion concentration changes during 3C fast discharging were calculated. All governing equations, respective boundary conditions, model parameters, and glossary of symbols for the electrochemical model are summarized in Tables S3–S6. To simulate the current density distribution as a function of perforation hole diameter, an electronic conduction model was developed using COMSOL Multiphysics 6.1 (COMSOL Inc., USA). Simulations were conducted for perforation diameters of 32.5, 75, 150, 300, and 600 μm. The electronic resistivity of the SiO_*x*_/AG electrode was obtained from experimental measurements, and a potential difference of 1 mV was applied as the boundary condition.

## Results and Discussion

### Electrode Design and Structural Characterizations

**Fig. 1:**
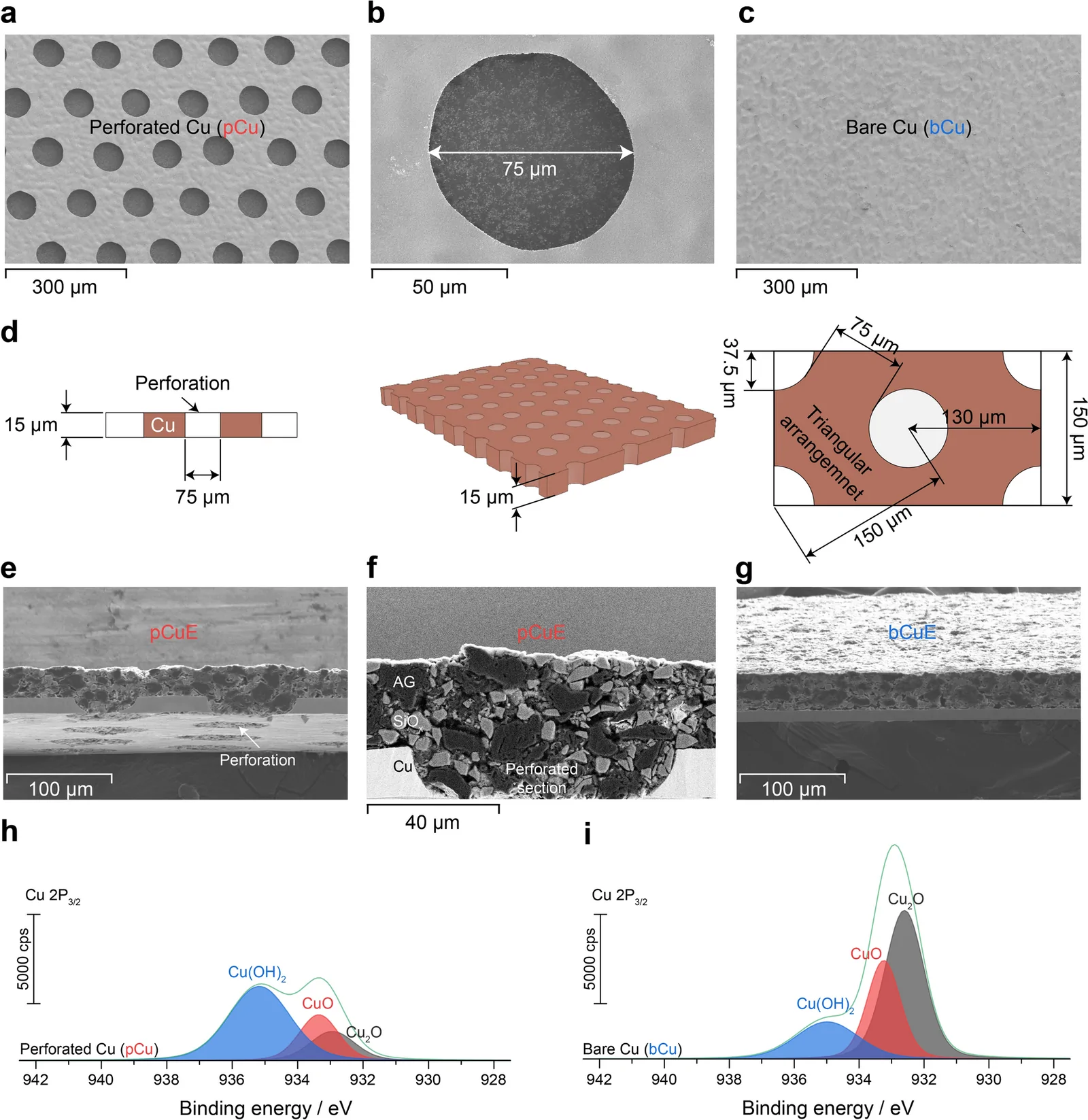
SEM images of **a**, **b** perforated Cu current collector (pCu) and **c** bare Cu current collector (bCu). **d** Schematic illustration of the detailed morphology and dimensions of the pCu. Cross-sectional SEM images of **e**, **f** pCu-adopted electrode (pCuE) and **g** bCu-adopted electrode (bCuE). X-ray photoelectron spectra of Cu 2_*P*3/2_ on **h** pCu and **i** bCu surface

The perforated Cu (pCu), which was fabricated via chemical etching, and the bare Cu (bCu) are presented in Fig. 1a–c. The SEM images clearly distinguish the micro-patterned perforations of the pCu from the non-perforated bCu. A more detailed schematic illustration of the perforation morphology, arrangement, and dimensions of the pCu is provided in Fig. 1d. Specifically, the thickness of the pCu is 15 μm, and the perforation holes have a diameter of 75 μm, arranged in a triangular pattern. Due to the presence of these microscale perforations, printed symbols placed behind the pCu remain visible, as demonstrated in Fig. S1.

Composite electrodes composed of the SiO_*x*_ and the AG were fabricated (Fig. 1e–g) by casting electrode slurry onto both the bCu and the pCu, with specific design parameters detailed in Table S1. The slurry consists of 31 wt% SiO_*x*_, 62 wt% AG, 4 wt% polyacrylic acid (PAA) binder, and 3 wt% Super P, resulting in a thinner electrode thickness thanks to the high SiO_*x*_ content. Particularly, the pCu-adopted electrode (pCuE) theoretically exhibits a 3 µm thinner electrode thickness compared to the bCu-adopted electrode (bCuE) because the perforated holes are filled with electrode material (Fig. S2). Cross-sectional SEM image in Fig. 1g and confocal microscopy image (Fig. S3) confirm uniform filling of the slurry within the patterned holes of the pCu. Cross-sectional EDX images (Fig. S4) provide further details on the morphologies of the pCuE and the bCuE, showing well-filled perforated holes with electrode material. Additionally, EDX elemental mapping provides the distribution of Si, C, and Cu within the electrodes.

The microscale holes in the pCuE create a distinctive interlocking structure (Fig. S5), resulting in a larger contact interface between the current collector and composite electrode compared to the bCuE. To assess the impact of this structure on electrochemical and mechanical properties, we analyzed the interface electronic resistivity and adhesion strength (Fig. S5). Using a multi-probe analyzer, we found that the interfacial electronic resistivity of the pCuE was ca. 20% of that of the bCuE, indicating better electron conductivity (Fig. S5a). Of note, the bulk resistivity of the composite electrodes was similar in both the pCuE and bCuE (Fig. S5b), which is reasonable given their identical composition, density, and mass loading. Although the bulk resistivity of the pCuE and bCuE was comparable, the presence of microscale perforated holes in the pCu might impede the efficient delivery of electrons to active materials in localized regions, potentially compromising the electrode’s rate and cycling performance. To examine this possibility, we employed magnetic field imaging (MFI), a technique that enables spatial mapping of current-induced magnetic fields across the electrode surface [48]. As shown in Fig. S6, a 3 A current was applied to both the bCu and pCu samples, and the resulting magnetic field distributions were measured along the x-, y-, and z-directions based on Ampère’s law. The measured field distributions revealed no discernible differences between the two samples, suggesting that the presence of perforations does not significantly perturb the current density distribution. Furthermore, electron conduction simulations implemented while varying the perforation diameter confirmed that current density remained uniform when the hole diameter was reduced to 75 μm or less—the same scale as our pCu used in this study—indicating minimal impact on electronic conduction. The surface and interfacial cutting analysis system (SAICAS), equipped with vertical and horizontal load and displacement sensors, precisely measures interface adhesion strength by cutting the sample vertically to the targeted interface and moving it horizontally (Fig. S8a). As shown in Fig. S8b, the pCuE exhibits a 10%–25% higher interfacial adhesion strength compared to the bCuE. This improvement is likely attributed to the increased interfacial area resulting from the interlocking structure. Moreover, since the perforation process was conducted via chemical etching with an acidic solution, the surface of the pCu can be modified to be more hydrophilic. In this regard, the pCu exhibits a significantly lower contact angle with deionized water, approximately 33 degrees, comparing to 64 degrees for the bCu (Fig. S9). Additionally, XPS analysis reveals that the pCu has a higher fraction of copper hydroxide relative to copper oxide (Fig. 1h, i). The abundant hydroxyl groups on the pCu surface likely enhance adhesion strength through additional hydrogen bonding with the PAA binder (Fig. S10) and improve slurry filling uniformity in the perforated holes by increased hydrophilicity.

**Fig. 2:**
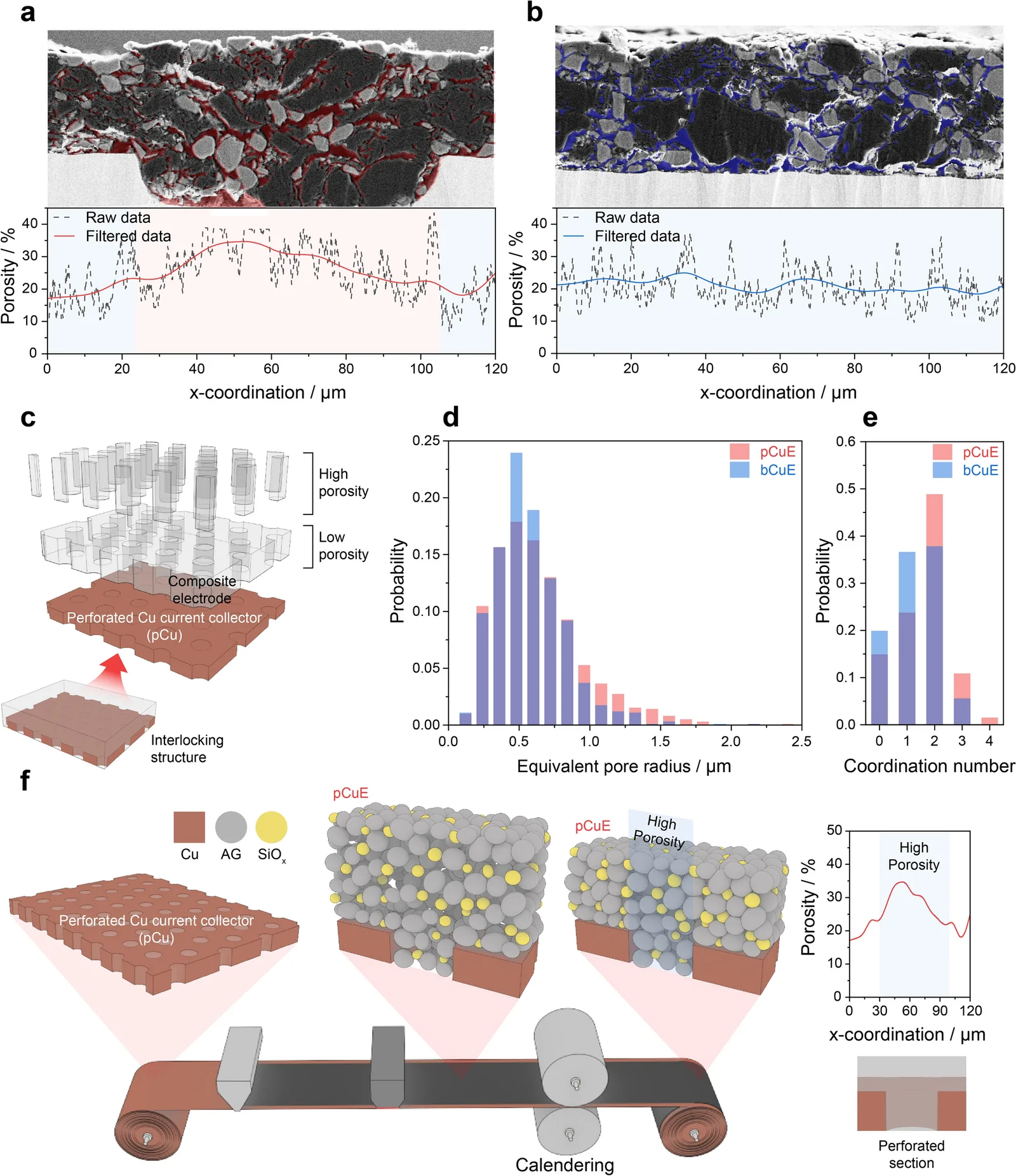
Pore domain segmented cross-sectional SEM images and porosity distribution along the x-direction for **a** pCuE and **b** bCuE. **c** Schematic illustration of the RAM structure in the pCuE. **d** Equivalent pore radius distribution and **e** coordination number distribution of the pCuE and the bCuE. **f** Schematic illustration of RAM structure formation during electrode fabrication process

The unique interlocking structure due to the micro-patterned holes of the pCu may affect the micropore structure of the pCuE. Therefore, we examined the porosity distribution utilizing 2D cross-sectional SEM images. Segmentation of the pore domain using gray value thresholding and marker-based watershed algorithms yielded the porosity distribution in Fig. 2a, b. Interestingly, in the SiO_*x*_/AG electrode, the perforated regions (PRs) of the pCuE exhibit 1.4 times higher porosity (ca. 28%) than the non-perforated regions (NPRs, ca. 19%), whereas the bCuE shows relatively uniform and intermediate porosity level (ca. 24%). This result clearly demonstrates the formation of the regularly arranged micropore structure or the RAM structure in the pCuE, alternating high- and low-porosity regions corresponding to the arrangement of perforation holes (Fig. 2c). Additionally, a pore network model analysis [46, 49] reveals that the pCuE has a larger equivalent pore radius (Fig. 2d, 0.68 μm for the pCuE and 0.60 μm for the bCuE) and a higher pore coordination number (Fig. 2e, 1.6 for the pCuE and 1.2 for the bCuE), indicating greater pore connectivity in the pCuE. Furthermore, since the electrode slurry uniformly filled the perforation holes due to the high hydrophilicity of the pCu, and the surface of the pCuE remains flat after drying process (as shown in confocal 3D image in Fig. S11), this implies that heterogeneous densification during the calendering process occurs depending on whether or not holes exist, as depicted in Fig. 2f.

In summary, the use of pCu induces the formation of the RAM structure in the electrode, characterized by alternating NPRs with relatively low porosity and PRs with higher porosity. Notably, the NPRs in the pCuE exhibit not only a larger equivalent pore size but also enhanced pore connectivity compared to those in the bCuE, indicating a more favorable microporous architecture. Given that both pCuE and bCuE were designed with identical total mass loading, the presence of electrode components filling the perforation holes inevitably leads to a reduction in the mass loading and thickness of the NPRs. Based on the geometrical parameters of the pCu (Fig. S12a) and assuming that the volume of active materials filling the PRs precisely compensates for the reduced material in the NPRs, the electrode thickness in the NPR is calculated to be approximately 3 μm thinner than that of the bCuE (Fig. S12b), resulting in an overall electrode thickness (excluding the current collector) of 30 μm, as previously shown in Fig. S2. Assuming a total mass loading of 4.95 mg cm^−2^ and an N/P ratio of 1.1 for the bCuE, the theoretical mass loading in the NPR and PR of the pCuE is estimated to be 4.50 and 6.75 mg cm^−2^, respectively (Fig. S12c). These correspond to local N/P ratios of 1.001 in the NPR and 1.50 in the PR, suggesting a potential risk of lithium plating in the NPR under aggressive cycling conditions due to its near-stoichiometric design. To improve the accuracy of this estimation, we further conducted image-based quantification using the cross-sectional SEM image of the pCuE (Fig. 2a). The calculated local mass loadings were 4.59 mg cm^−2^ in the NPR and 6.62 mg cm^−2^ in the PR, corresponding to local N/P ratios of 1.02 and 1.47, respectively (Fig. S12d). While these values confirm that lithium plating is unlikely across the electrode, the analysis highlights the importance of carefully considering local N/P ratio distributions—particularly in the NPR—during electrode design. Finally, based on the measured mass loading and porosity values (19% in NPR and 28% in PR), the local electrode densities were estimated to be 1.61 g cm^−3^ in the NPR and 1.42 g cm^−3^ in the PR.

### Electrochemical Performances of pCuE

**Fig. 3:**
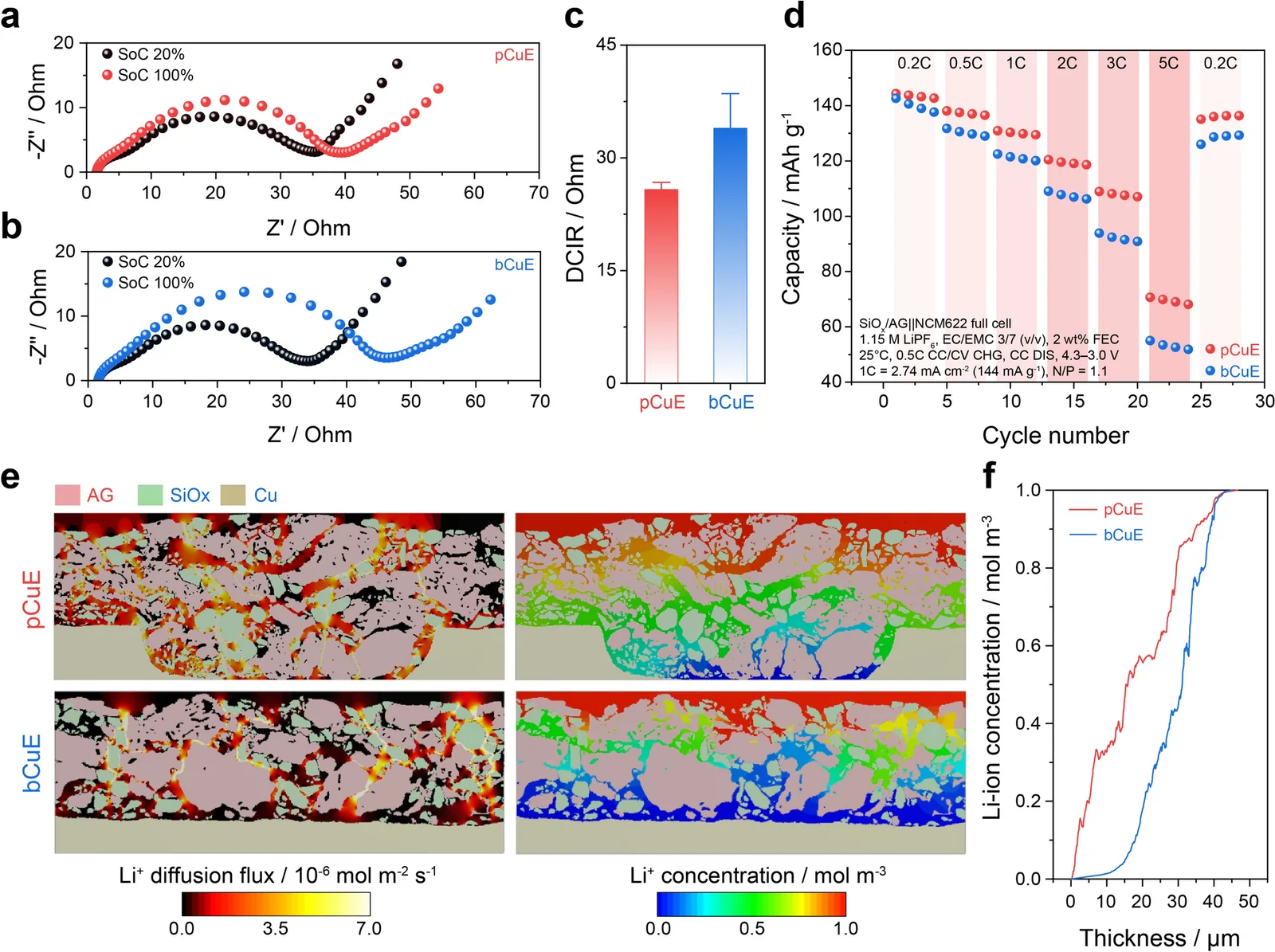
Nyquist plots of electrochemical impedance spectra measured at 20% and 100% SoC after precycling for **a** pCuE half-cells and **b** bCuE half-cells. **c** SoC-averaged direct current internal resistance (DCIR) of the pCuE and the bCuE half-cells estimated using the hybrid pulse power characterization (HPPC) protocol. **d** Rate test results of the pCuE and the bCuE full cells. **e** 2D color maps of lithium-ion diffusion flux and lithium-ion concentration for the pCuE and the bCuE calculated from two-dimensional lithium-ion diffusion simulations. **f** Lithium-ion concentration distribution curve as a function of electrode thickness

Figures 3a, b and S13a, b present the EIS spectra for the pCuE and the bCuE half-cells, measured from 10 to 100% SOC in 10% increments after precycling. The corresponding precycling voltage profiles of both half-cells are provided in Fig. S14 for reference. Resistance analysis using the equivalent circuit model (Fig. S13c) shows that charge transfer resistance (R_ct_) is lower in the pCuE half-cell than in the bCuE half-cell (Fig. S13d). Additionally, hybrid pulse power characterization (HPPC) results indicate that the direct current internal resistance (DCIR) of the pCuE half-cell is 24% lower than that of the bCuE half-cell (Figs. 3c and S15), implying superior power characteristics of the pCuE. Therefore, to further evaluate the rate performance of the pCuE, full cells were subsequently assembled with LiNi_0.6_Co_0.2_Mn_0.2_O_2_ (NCM622) cathode of electrode design specified in Table S2. (For reference, precycling voltage profiles and rate performance of the NCM622 half-cell are presented in Fig. S16.)

In full cells, the pCuE exhibited lower interfacial resistance compared to the bCuE, as confirmed by EIS measurements (Fig. S17). Consistently, rate capability tests demonstrated the superior performance of the pCuE full cell (Fig. 3d). To elucidate the role of the RAM structure in enhancing rate capability, two-dimensional diffusion simulations were performed using segmented cross-sectional SEM images (Fig. S18). The simulation results reveal that lithium-ion diffusion in the bCuE is highly localized, with elevated flux concentrated in limited regions. In contrast, the PRs of the pCuE exhibit a more uniform diffusion flux (Fig. 3e). Consequently, lithium-ion concentration in the PRs remains relatively high even at the bottom of the perforated holes, whereas in the bCuE, the concentration progressively decreases from the top to the bottom of the electrode, resulting in ion depletion near the current collector (Fig. 3e, f). These results can be attributed to the presence of highly porous domains (HPds) within the PRs, which are likely to facilitate more favorable ion percolation pathways, as indicated by the preceding pore network analysis. However, it is noteworthy that relatively high lithium-ion concentrations were also observed in the low-porosity domains (LPds) located in the neighboring NPRs. This suggests that the HPd not only enhances ion transport locally within the PRs, but may also promote lithium-ion diffusion in adjacent LPd regions, despite their intrinsically lower porosity. Although further investigation is necessary, these findings imply that the RAM structure in the pCuE may support more uniform and efficient lithium-ion transport throughout the entire electrode.

**Fig. 4:**
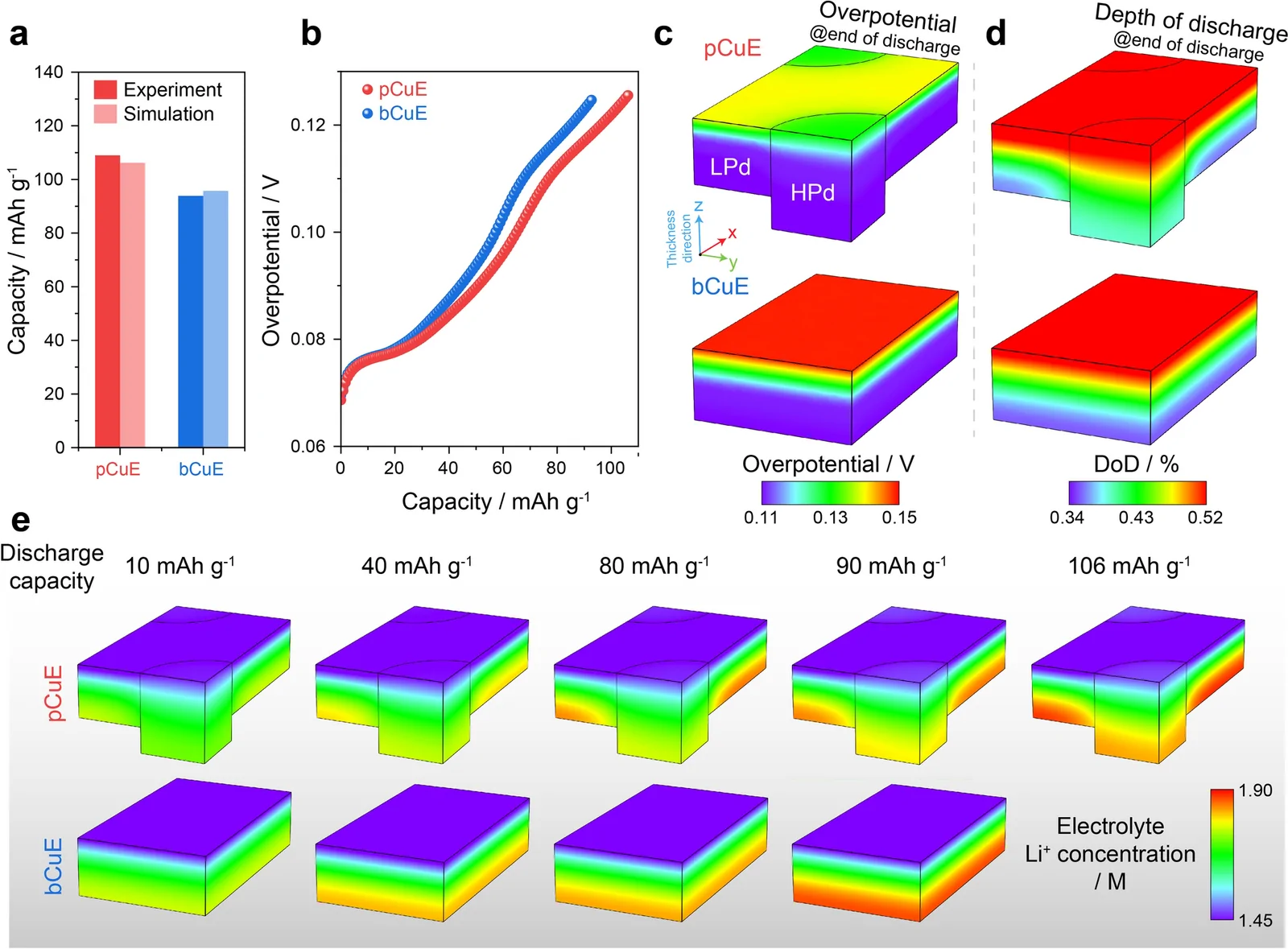
**a** 3C discharge capacity of the pCuE and the bCuE full cells from experiments and microstructure-resolved electrochemical simulations. **b** Overpotential change as a function of discharge capacity estimated from electrochemical simulations. **c** 3D color maps of overpotential and **d** depth of discharge (DoD) for the pCuE and the bCuE at the end of the 3C discharge process. **e** Spatial distribution of lithium ions in the electrolyte domain as a function of discharge capacity

To further investigate the impact of the RAM structure on rate performance and to validate the preceding assumptions, a pseudo-four-dimensional (P4D) electrochemical modeling and simulation-based analysis [47, 50] was conducted. The detailed modeling domain and geometry are illustrated in Fig. S19. The 3C fast discharge simulation results in a capacity similar to that observed in the experiment (Fig. 4a), and the simulated voltage profiles qualitatively captured the trends observed in the experimentally measured profiles of both pCuE and bCuE (Fig. S20), thereby confirming the basic validity of the electrochemical model. As shown in Fig. 4b, the pCuE exhibited a lower overpotential compared to the bCuE, resulting in a higher discharge capacity. This behavior was further examined using 3D color maps (Fig. 4c, d). At the end of discharge, the pCuE displays reduced overpotential than the bCuE, particularly in the HPds, compared to the LPds. This is attributed to the higher porosity and improved pore connectivity of the HPd. In contrast, the bCuE exhibited a substantial overpotential, especially at the top of the electrode. Moreover, the depth of discharge (DoD) in the HPd was higher than in the LPd, and even within the same LPd, regions adjacent to the HPd showed a higher DoD. This suggests that the presence of the HPd can promote lithium-ion diffusion into the LPd, consistent with the earlier discussion in Fig. 3e. In contrast, the bCuE exhibited a lower DoD across all domains compared to the pCuE. Additionally, we analyzed changes in lithium-ion concentration in the electrolyte domain. As shown in Fig. S21a, the pCuE exhibited a lower degree of lithium-ion accumulation in the electrolyte compared to the bCuE, indicating that the superior pore structure in the pCuE can suppress concentration polarization even at high C-rates, as depicted in Fig. S21b. The spatial distribution of lithium-ion in Fig. 4e further confirms that the pCuE maintains a smaller concentration gradient along the electrode depth than the bCuE due to the presence of HPd. In summary, from a geometrical perspective, the perforation holes in the pCuE not only shorten the ion transport distance in the NPR (or LPd) by reducing the local electrode thickness to 30 μm, but also increase the local electrolyte volume within the PR (or HPd), thereby alleviating lithium-ion concentration gradients. Furthermore, the well-developed pore network in the HPd promotes more efficient ion transport and helps mitigate lithium-ion accumulation even in adjacent LPd regions. Together with the experimental observations, these simulation results highlight the synergistic lithium-ion transport behavior enabled by the RAM architecture in the pCuE.

**Fig. 5:**
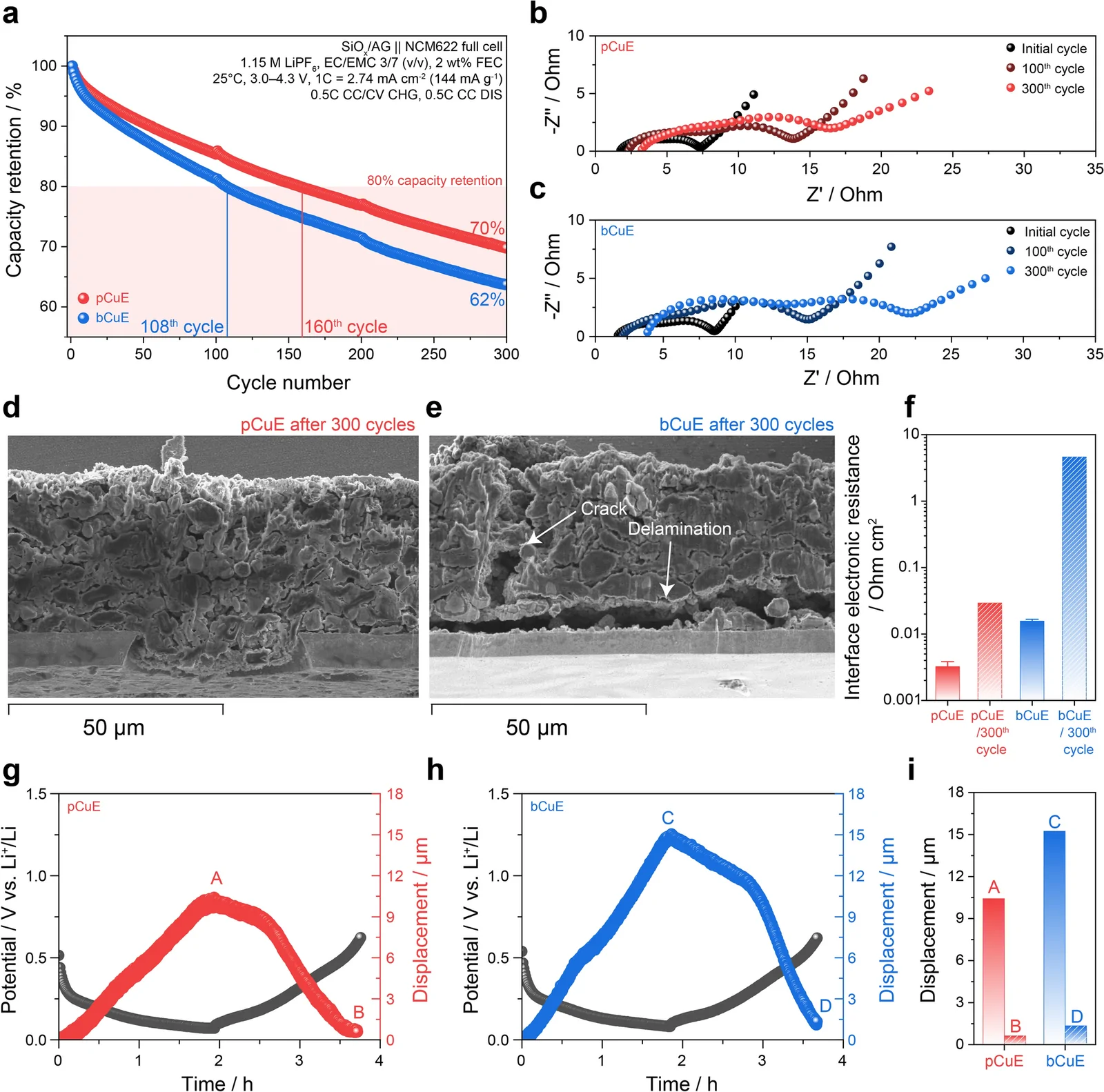
**a** Long-term cycling performance of the pCuE and the bCuE full cells. Nyquist plot of electrochemical impedance spectra measured before cycling, after 100 cycles, and after 300 cycles for **b** pCuE and **c** bCuE full cells in the fully discharge state. Postmortem cross-sectional SEM images after 300 cycles for **d** pCuE and **e** bCuE. **f** Interface electronic resistivity of the pCuE and the bCuE before and after 300 cycles. Potential-time and electrode thickness displacement–time co-plotted curves obtained from electrochemical dilatometry measurements of **g** pCuE and **h** bCuE three-electrode pouch cells. **i** Displacement at the end of the charge and discharge process for the pCuE and the bCuE

In the rate test (Fig. 3d), after varying the applied current from 0.2C to 5C and then back to 0.2C, the pCuE retains higher capacity compared to the bCuE. To further clarify differences in capacity retention, a 0.5C cycle test was conducted (Figs. 5a and S22) after precycling. Similar to the rate test results, the pCuE full cell shows a more stable capacity retention curve, reaching 80% of its initial capacity at the 160th cycle, whereas the bCuE full cell drops to 80% capacity retention at the 108th cycle. The cycling stability achieved by the pCuE—enabled by the use of the perforated Cu current collector—is comparable to, or even exceeds, that reported in recent state-of-the-art studies employing surface-modified silicon-based active materials (Table S7), despite the high SiO_*x*_ content (30 wt% relative to the active material) and the use of non-surface-treated commercial SiO_*x*_ in this work [33, 51–61]. EIS analysis before and after cycling indicates that the resistance of the bCuE full cell increases more rapidly than that of the pCuE full cell (Figs. 5b, c and S23). Postmortem analysis after 300 cycles reveals no significant delamination between the current collector and composite electrode in the pCuE (Fig. 5d), while the bCuE shows noticeable electrode delamination and cracks. Interface electronic resistivity measurement also demonstrates that the resistivity of the pCuE increases by an order of magnitude after cycling, whereas that of the bCuE increases by two orders, indirectly indicating less delamination in the pCuE (Fig. 5f). Since continuous volume change of active material leads to contact loss, dead particle formation, electrode delamination, and cracks, one of the primary causes of degradation in high-content SiO_*x*_ electrodes is mechanical degradation. To assess these microstructural changes, operando electrochemical dilatometry was conducted to measure thickness changes in three-electrode pouch-type full cells during charging and discharging (Fig. S24). The thickness of the bCuE full cells increases rapidly from the start of charging, reaching a thickness displacement of approximately 15 µm by the end of the charge process, compared to ca. 10 µm for the pCuE full cells (Fig. 5g, h). After subsequent discharge, the thickness of the bCuE full cells remains 1.34 µm higher than the initial thickness, whereas the pCuE full cell is only 0.63 µm higher, indicating over twice the irreversible thickness change in the bCuE full cell (Fig. 5i). The superior electrochemo-mechanical reversibility of the pCuE full cells is likely due to better interfacial adhesion, a relatively lower thickness above the current collector surface, and the presence of high-porosity regions, which act as buffer spaces for volume changes.

**Fig. 6:**
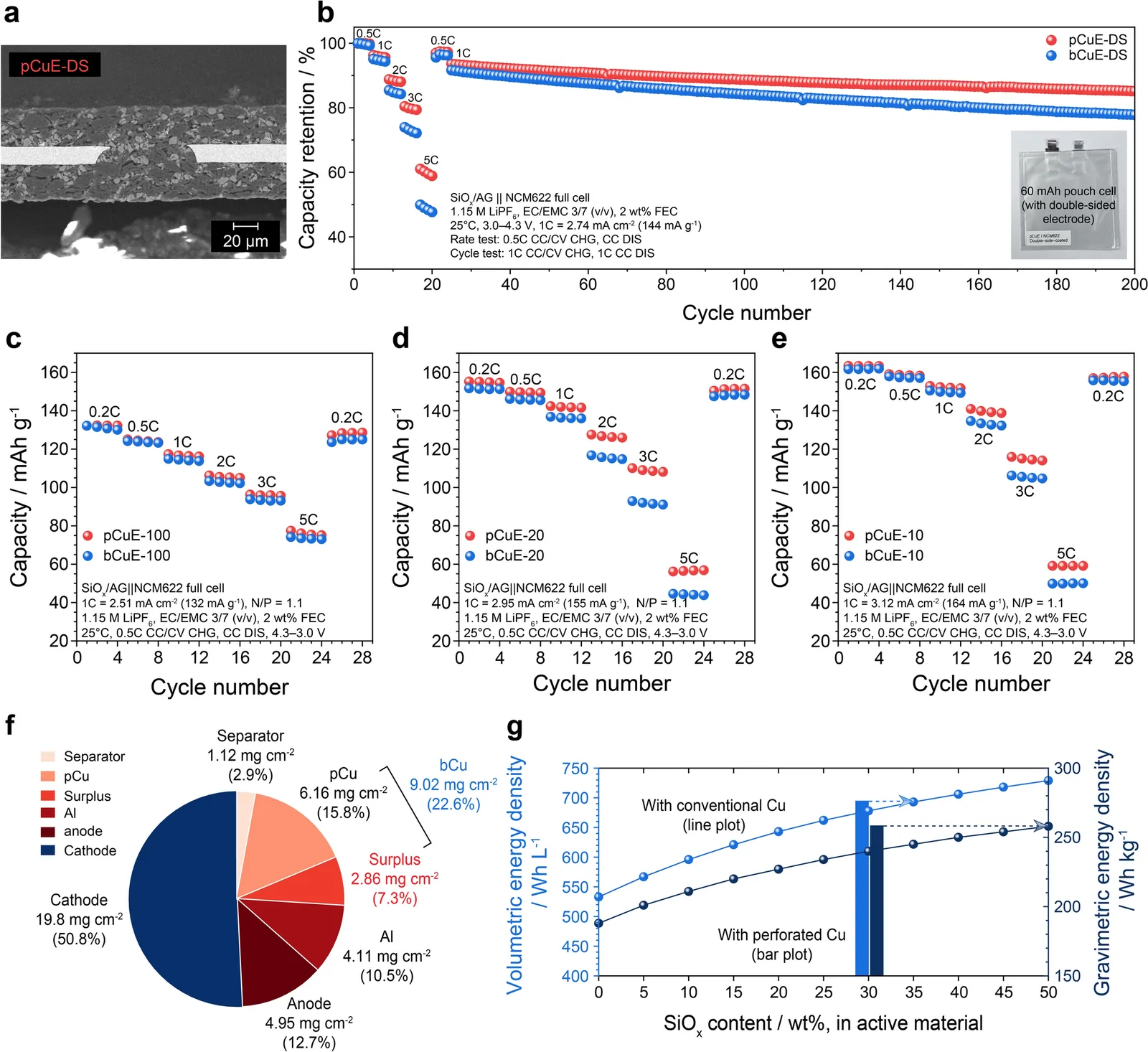
**a** Cross-sectional SEM image of the double-sided pCu electrode (pCuE-DS). **b** Capacity retention curves of the pCuE-DS and bCuE-DS pouch-type full cells during rate tests and 1C cycling. **c** Rate performance of full cells with SiO_*x*_-only electrodes (comprising 100 wt% SiO_*x*_ within the active material) using pCu (pCuE-100) and bCu (bCuE-100). **d** Rate performance of full cells with SiO_*x*_/AG composite electrodes containing 20 wt% SiO_*x*_ in the active material using pCu (pCuE-20) and bCu (bCuE-20). **e** Rate performance of full cells with SiO_*x*_/AG composite electrodes containing 10 wt% SiO_*x*_ in the active material using pCu (pCuE-10) and bCu (bCuE-10). **f** Mass loading and the corresponding percentage of each cell component (cathode, anode, separator, Cu foil, and Al foil). **g** Volumetric and gravimetric energy densities of the bCuE cell as a function of SiO_*x*_ content in the active material, with comparison to the pCuE cell containing 30 wt% SiO_*x*_

To evaluate the practical applicability of the pCuE incorporating the RAM structure in commercial LIBs, we fabricated a double-sided SiO_*x*_/AG electrode using a perforated Cu current collector (pCuE-DS). During the initial coating process, no slurry penetration or leakage through the perforation holes to the opposite side was observed (Fig. S25a). After drying the first coated layer, a second slurry layer was successfully applied to the opposite side, yielding a double-sided electrode as shown in Fig. S25b, c. Cross-sectional SEM images (Fig. 6a) confirmed uniform coating and comparable thicknesses on both sides, indicating successful fabrication. In addition, X-ray computed tomography (XCT) was employed to non-destructively assess the internal structure of the pCuE-DS across a larger electrode area. As shown in Fig. S26, the XCT results revealed no significant defects and confirmed that electrode materials were well infiltrated into the perforated regions. Using the fabricated pCuE-DS and bCuE-DS electrodes, we assembled two-stack pouch-type full cells with a nominal capacity of 60 mAh. (Precycling voltage profiles are provided in Fig. S27.) Rate capability tests (0.5C to 5C and back to 0.5C) and long-term cycling at 1C confirmed that the pCuE-DS cell consistently outperformed the bCuE-DS counterpart in both rate and cycling performance. These results strongly support the practical feasibility of implementing the pCu-based RAM structure in commercial cells employing double-sided electrodes.

To further evaluate the effectiveness of pore regulation by pCu across different SiO_*x*_ contents, we extended the analysis to a series of electrodes with varying active material compositions: 100 wt% SiO_*x*_ (pCuE-100, bCuE-100), 20 wt% SiO_*x*_ (pCuE-20, bCuE-20), and 10 wt% SiO_*x*_ (pCuE-10, bCuE-10). Detailed electrode morphology and design parameters are presented in Fig. S28. Electrochemical impedance spectroscopy (EIS) of symmetric cells (Fig. S29) was used to quantify the ionic resistance (R_ion_) and ionic tortuosity, the latter of which reflects the complexity of ion transport pathways [62, 63]. Across all electrode compositions, R_ion_ was consistently lower in pCuE samples than in their bCuE counterparts. Interestingly, the extent of this reduction varied with electrode thickness: when the electrodes were either very thin (e.g., pCuE-100, thickness = 16 μm) or relatively thick (e.g., pCuE-10, thickness = 53 μm), the difference was less pronounced. This suggests that pore modulation effects induced by perforation—likely arising from non-uniform densification during calendering—are most effective at intermediate thicknesses. Consistent with the resistance trends, calculated ionic tortuosity values were also lower in all pCuEs than in their corresponding bCuEs (Fig. S29f), confirming the beneficial role of the perforated current collector in regulating pore structure. The equivalent circuit model and the tortuosity calculation methods are provided in Fig. S29g–i. Furthermore, rate performance was assessed for each electrode design using full cells under various discharge currents (0.2C to 5C, and CC mode). As shown in Figs. 6c–e and S30, the results were consistent with the impedance and tortuosity analyses: cells utilizing pCuE electrodes exhibited comparable or superior rate capability relative to those using bCuE.

Beyond electrochemical performance, the introduction of perforated holes also reduces the mass of the current collector, contributing to improved cell-level energy density. Assuming a 10 µm thickness, the areal mass of the pCu was 6.16 mg cm^−2^, approximately 32% lower than that of the bCu (9.02 mg cm^−2^). The mass contributions of each component—including cathode, anode, separator, Cu foil, and Al foil—were measured and summarized in Fig. 6f. In the bCu-based cell, the Cu current collector accounted for 22.6% of the total cell mass, whereas in the pCu-based cell, this was reduced to 15.8%, resulting in a net mass reduction or surplus of 2.86 mg cm^−2^ (equivalent to 7.3%). Consequently, the gravimetric energy density increased from 240 (bCuE cell) to 258 Wh kg^−1^ (pCuE cell), while the volumetric energy density improved from 678 to 694 Wh L^−1^. These improvements correspond to gains of 7.5% and 2.3%, respectively, attributable to perforation-induced mass and thickness reductions. The detailed energy density calculation methods are provided in Note S1. Notably, when compared with the effect of increasing SiO_*x*_ content, the use of pCu alone yielded a gravimetric energy density gain equivalent to increasing the SiO_*x*_ content from 30 wt% to 50 wt%, and a volumetric energy density gain comparable to a 5 wt% increase (Fig. 6g). These findings demonstrate that perforated Cu can serve as an effective design strategy to enhance energy density in LIBs.

While increasing the degree of perforation could potentially further improve energy density, the mechanical integrity of the pCu must be considered, particularly for industrial-scale applications. Excessive perforation may compromise mechanical robustness during calendaring or jelly-roll winding, which involve substantial mechanical stress. To assess this, tensile strength tests were conducted using a universal testing machine (Fig. S31). The measured tensile strength of pCu was 74.9 MPa, approximately 35.4% of the bCu foil (212.5 MPa, 10 μm thick). Although lower than conventional Al current collectors (119.5 MPa, 15 μm) and even commercial PE separators (103.9 MPa, 14 μm), this value remains significantly above the tensile stress typically encountered in roll-to-roll (R2R) processing. Specifically, based on literature values, web tension in R2R lines ranges from 30 to 200 N [64–66], translating to an applied stress of ~ 22 MPa (200N) for a 600 mm-wide, 10 μm-thick Cu foil—well below the measured strength of pCu. This indicates that the pCu is mechanically robust enough to withstand the demands of industrial processing, including continuous roll handling, calendering, and winding.

## Conclusions

In this study, we demonstrate that the pore structure within composite electrodes can be engineered into the RAM structure using the pCu without additional post-processing. The RAM structure in the pCuE significantly enhances the rate capability of high-content SiO_*x*_/AG anodes. To elucidate the influence of pore microstructure on lithium-ion transport, we conduct microstructure resolved two-dimensional diffusion simulations and pseudo-four-dimensional (P4D) electrochemical simulations. The results reveal that the regularly arranged HPd within the RAM structure facilitates lithium-ion diffusion into LPd, effectively mitigating lithium-ion concentration polarization and enabling uniform ion distribution. Furthermore, the hydroxyl group-rich surface of pCu, the unique interlocking structure of pCuE, the reduced electrode thickness under identical design conditions, and the distinctive RAM architecture collectively contribute to suppressing mechanical degradation and improving the long-term cyclability of high-content SiO_*x*_-based LIBs.

## Supplementary Information

Below is the link to the electronic supplementary material.

Supplementary file1 (DOCX 8978 KB)
