# Supplementary material for: Regularly Arranged Micropore Architecture Enables Efficient Lithium-Ion Transport in SiOx/Artificial Graphite Composite Electrode
Source: Nanomicro Lett. 2025 Oct 9;18:75. doi: 10.1007/s40820-025-01929-4 (PMC12508359; doi:10.1007/s40820-025-01929-4)
Supplement: Supplementary file 1 — Supplementary file1 (DOCX 8978 KB) [file 40820_2025_1929_MOESM1_ESM.docx]

Supporting Information for

Regularly Arranged Micropore Architecture Enables Efficient Lithium-Ion Transport in SiO_x_/Artificial Graphite Composite Electrode

Jaejin Lim^1,†^, Dongyoon Kang^1,†^, Cheol Bak^2,†^, Seungyeop Choi^1^, Mingyu Lee^3^, Hongkyung Lee^3,4,**^, Yong Min Lee^1,2,3*^

^1^Department of Chemical and Biomolecular Engineering, Yonsei University, 50 Yonsei-ro, Seodaemun-gu, Seoul 03722, Republic of Korea
^2^Department of Energy Science and Engineering, Daegu Gyeongbuk Institute of Science and Technology (DGIST), Daegu 42988, Republic of Korea

^3^Department of Battery Engineering, Yonsei University, 50 Yonsei-ro, Seodaemun-gu, Seoul 03722, Republic of Korea

^4^Department of Materials Science and Engineering, Yonsei University, 50 Yonsei-ro, Seodaemun-gu, Seoul 03722, Republic of Korea

† Jaejin Lim, Dongyoon Kang, and Cheol Bak contributed equally to this work.

*Corresponding authors. E-mail: [yongmin@yonsei.ac.kr](mailto:yongmin@yonsei.ac.kr) (Yong Min Lee); [hongkyung.lee@yonsei.ac.kr](mailto:hongkyung.lee@yonsei.ac.kr) (Hongkyung Lee)

**S1 Supplementary Figures and Tables**


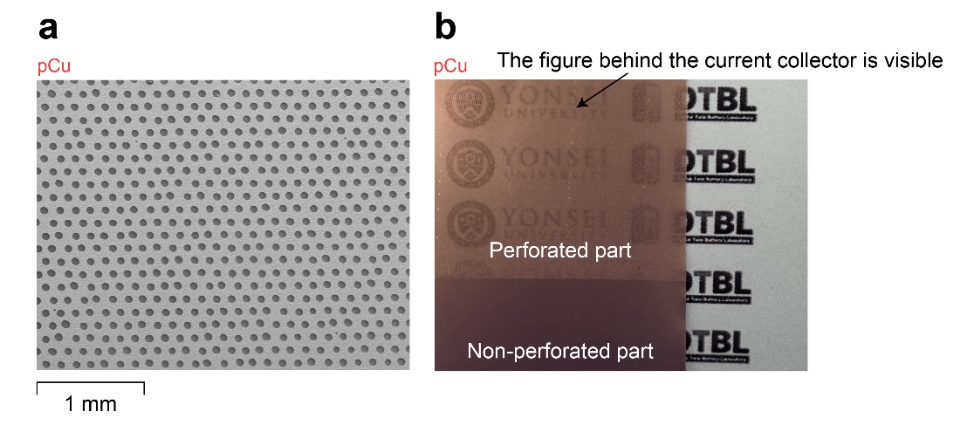


**Fig. S1** **a** SEM image and **b** optical image of the perforated Cu current collector (pCu). Due to the micrometer-scale perforation holes, the figure behind the pCu is visible


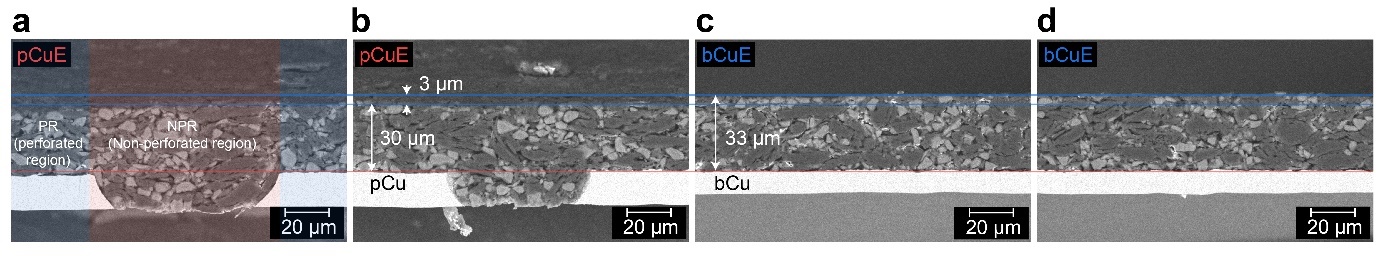


**Fig. S2** Cross-sectional SEM images of **a, b** the pCuE and **c, d** bCuE used for electrode thickness comparison


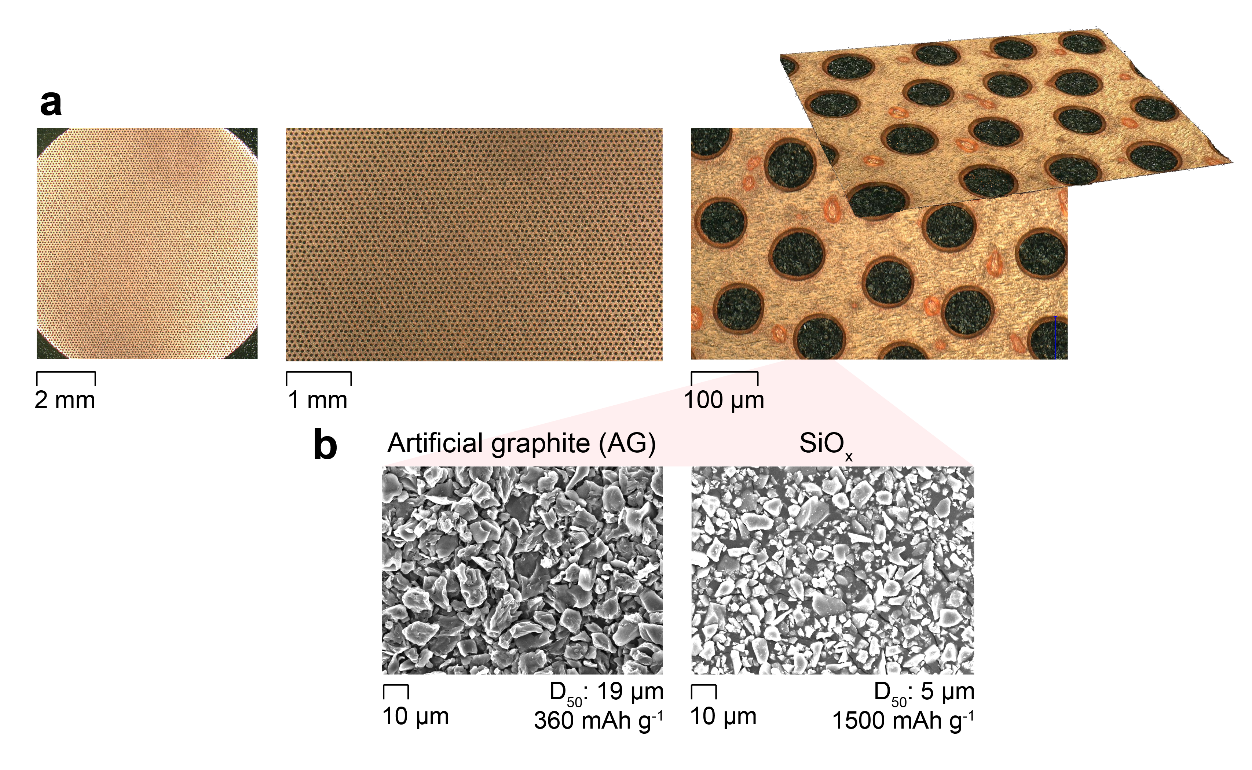


**Fig. S3 a** Confocal microscopy image of the pCu-adopted electrode (pCuE), showing the opposite side of slurry coating layer. **b** SEM image of artificial graphite and SiO_x_ active materials


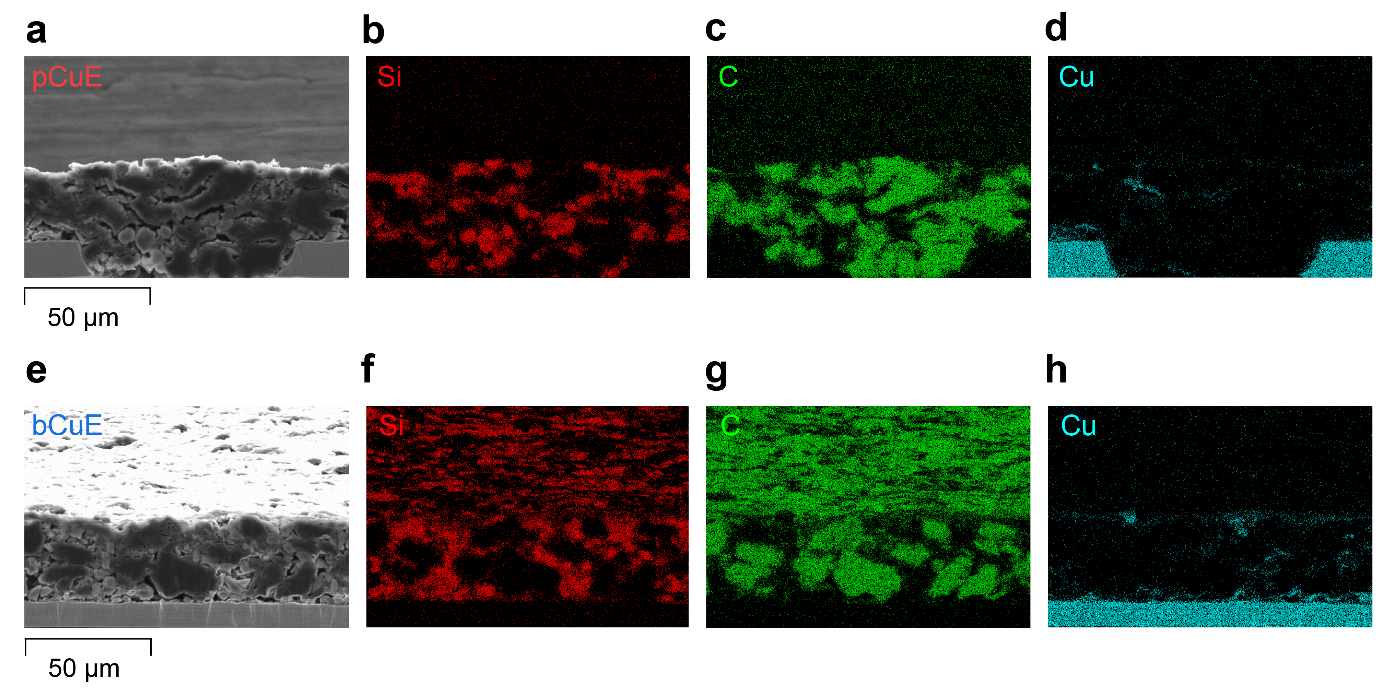


**Fig. S4** Cross-sectional SEM/EDX images of the pCuE and the bCuE: **a** Cross-sectional SEM image of the pCuE, with EDX element mapping for **b** Si, **c** C, and **d** Cu; **e** Cross-sectional SEM image of the bCuE, with EDX element mapping for **f** Si, **g** C, and **h** Cu

**
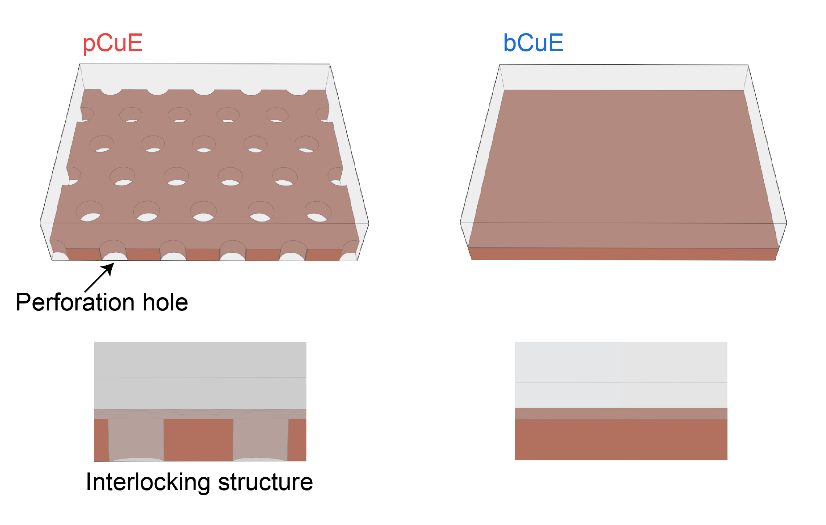
**

**Fig. S5** Schematic illustration of the pCuE and the bCuE


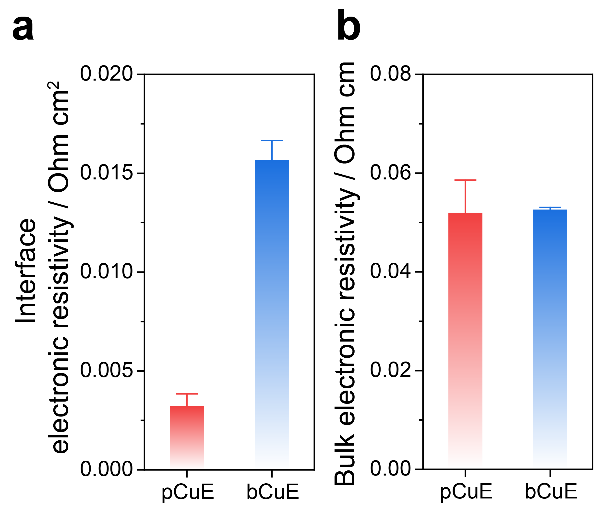


**Fig. S5 a** Bulk electronic resistivity and **b** interface (current collector and composite electrode) electronic resistivity of the pCuE and the bCuE measured using multi-probe electrode resistance analyzer


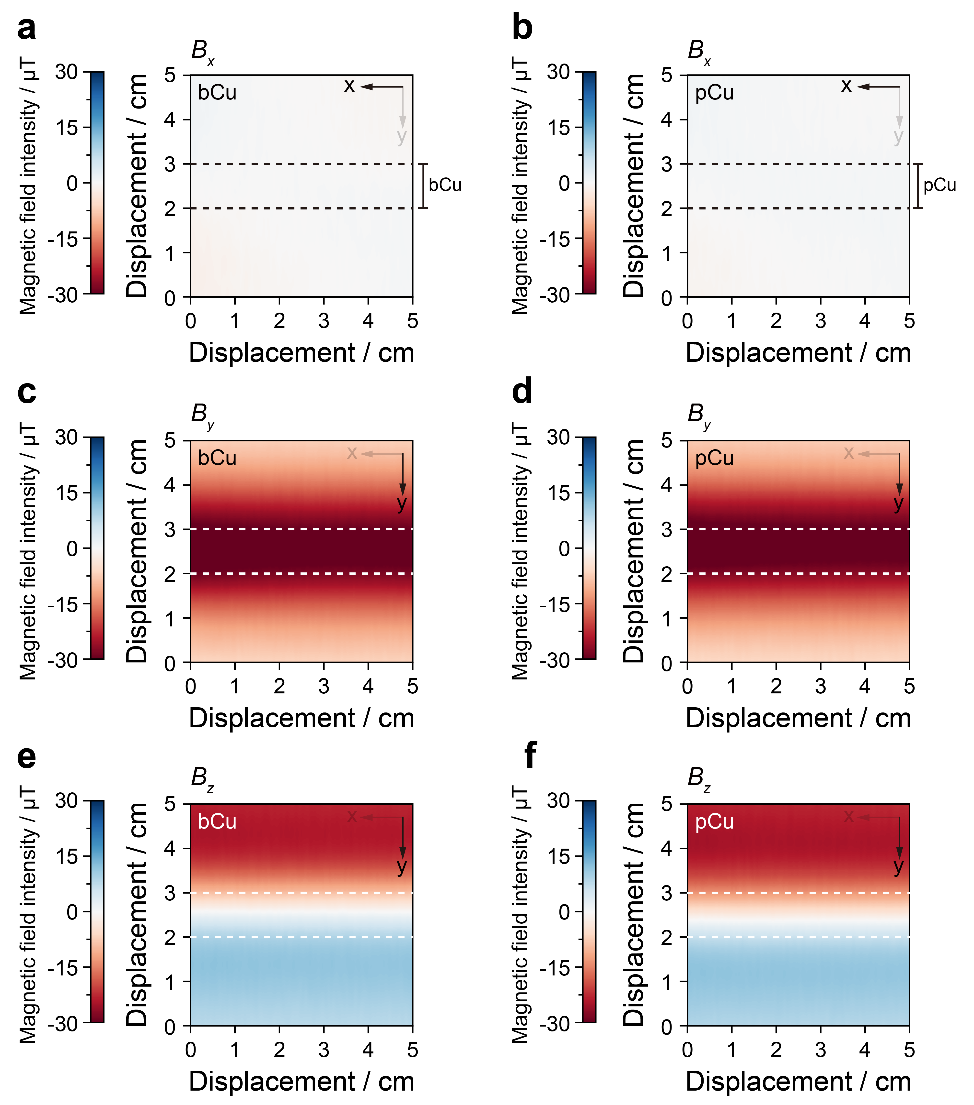


**Fig. S6** **a, b** Bx, **c, d** By, and **e, f** Bz magnetic field imaging (MFI) maps for the bCu and pCu foils, respectively. The foils are placed along the dashed lines for reference


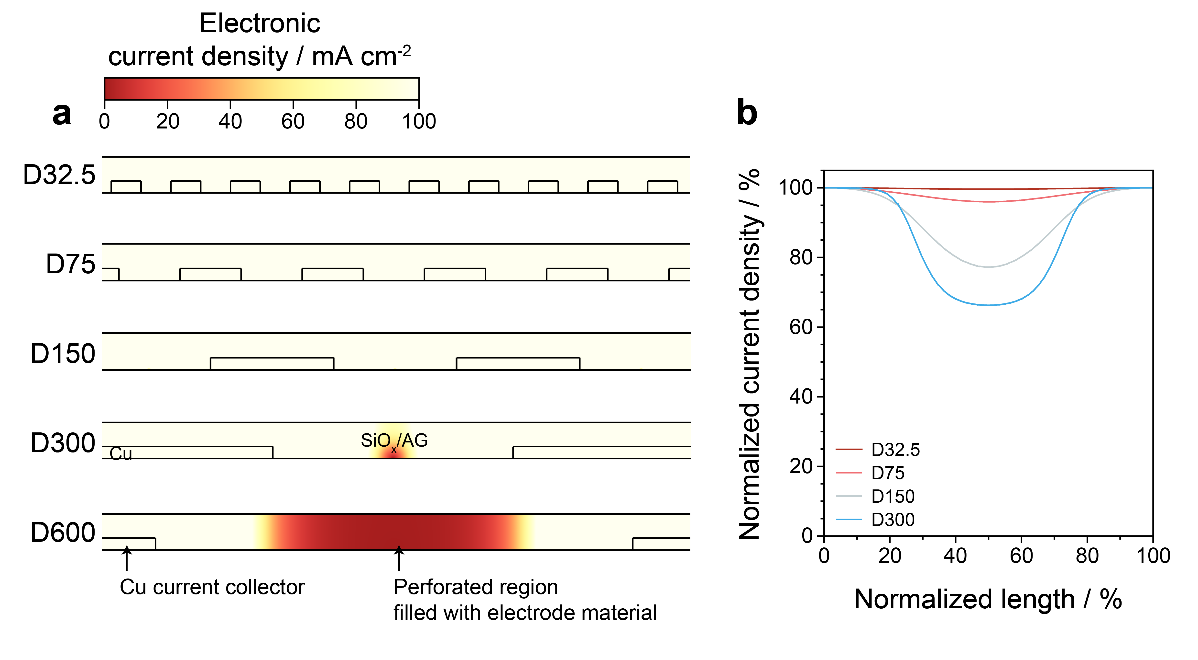


**Fig. S7** **a** Electrode current density distribution obtained from electron conduction simulation as a function of the perforation hole diameter. **b** Normalized current density distribution of the SiO_x_/AG electrode located in the perforated region


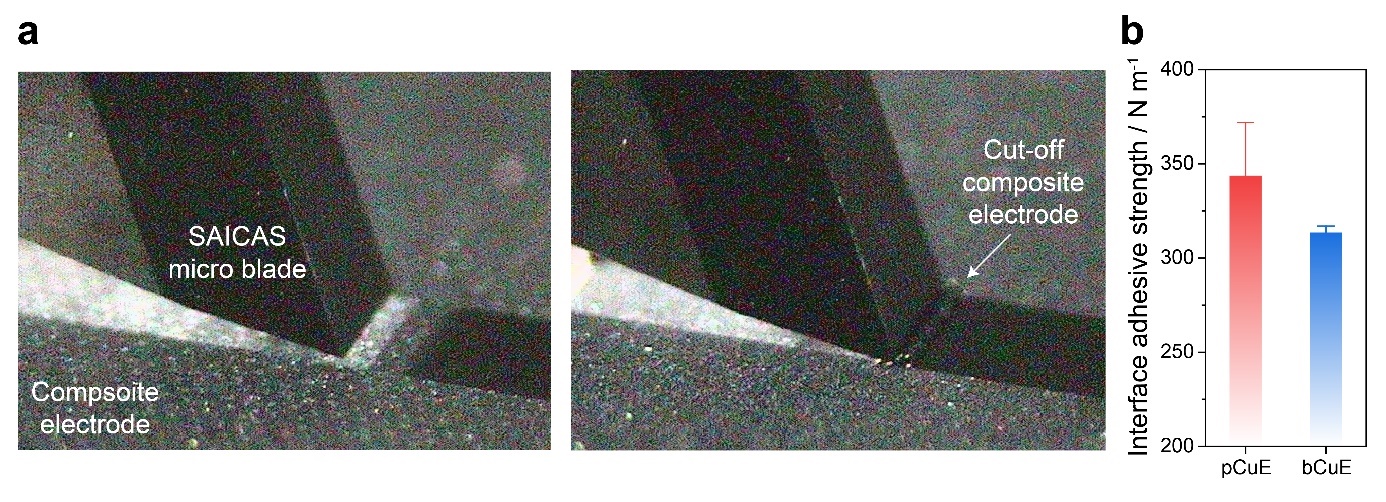


**Fig. S8 a** Optical microscopy images of the SAICAS micro-blade and the electrode sample before and after measurement. **b** Interface adhesion strength between current collector and composite electrode for the pCuE and the bCuE

**
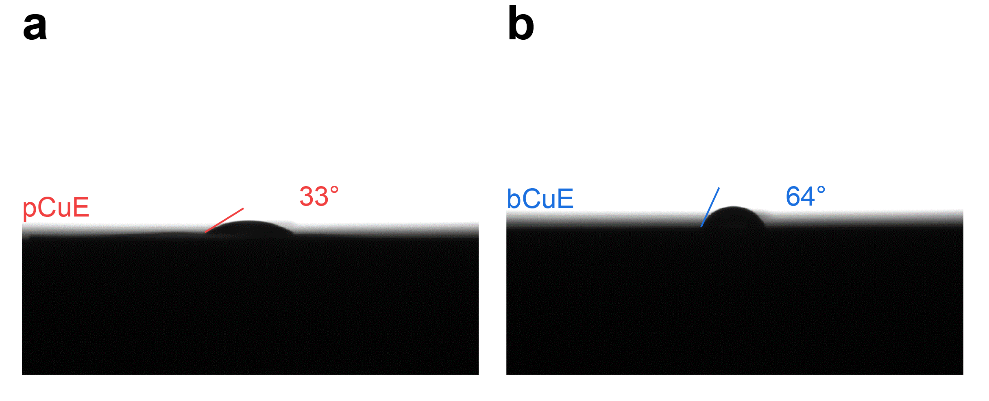
**

**Fig. S9** Contact angle of **a** the pCu and **b** the bCu with deionized water

**
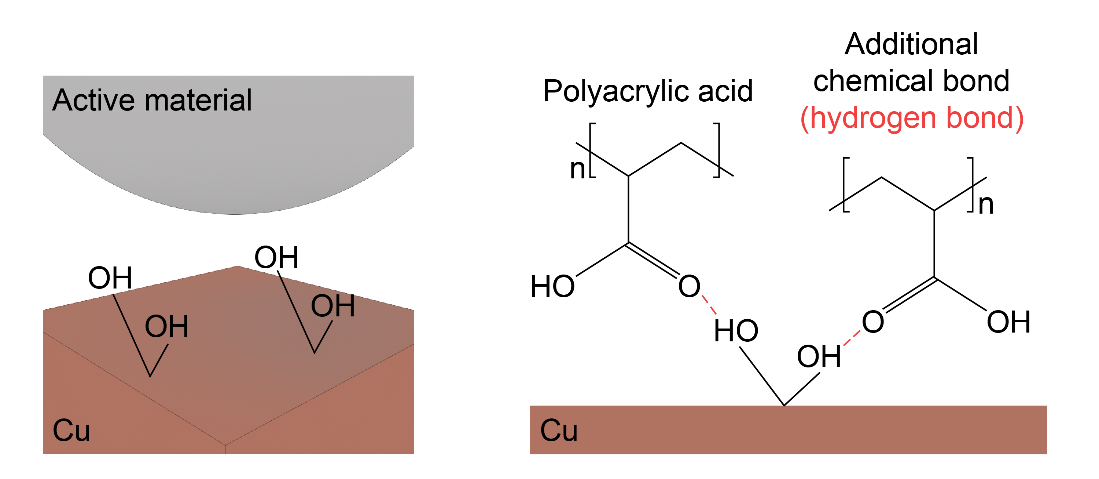
**

**Fig. S10** Schematic illustration of additional hydrogen bonding between hydroxyl group on the pCu and the PAA binder


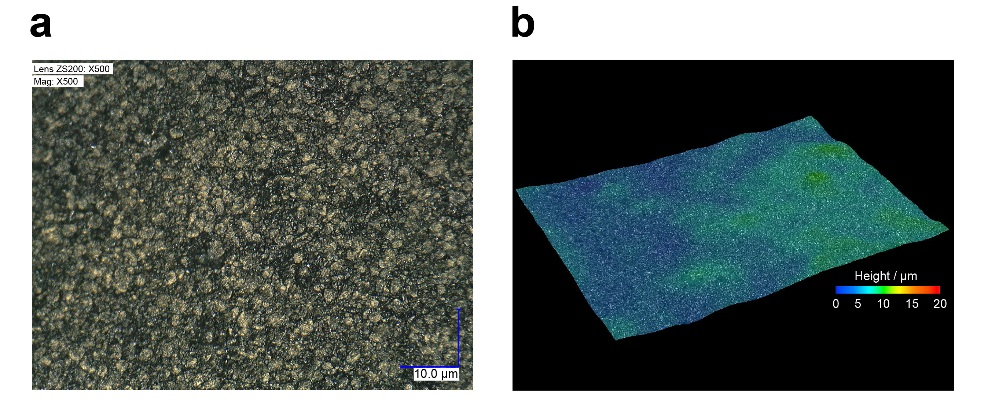


**Fig. S11 a** Confocal microscopy image of the surface of the pCuE. **b** Surface profile of the corresponding pCuE surface


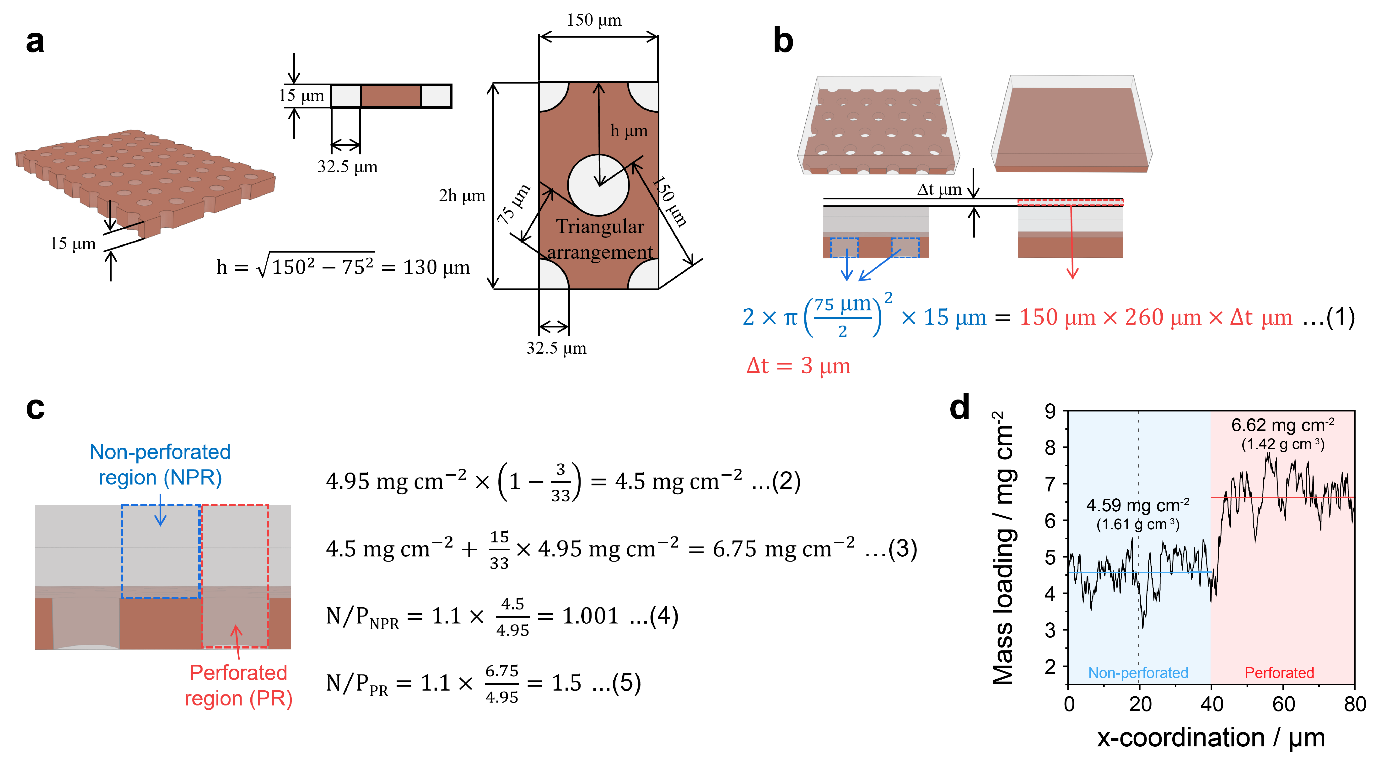


**Fig. S12 a** Detailed geometry of the pCu. Schematic illustration of the calculation procedure for **b** the electrode thickness reduction in the non-perforated region (NPR) and **c** the local N/P ratio in the NPR and perforated region (PR) of the pCuE. **d** Comparison of mass loading and the corresponding electrode density in the NPR and PR


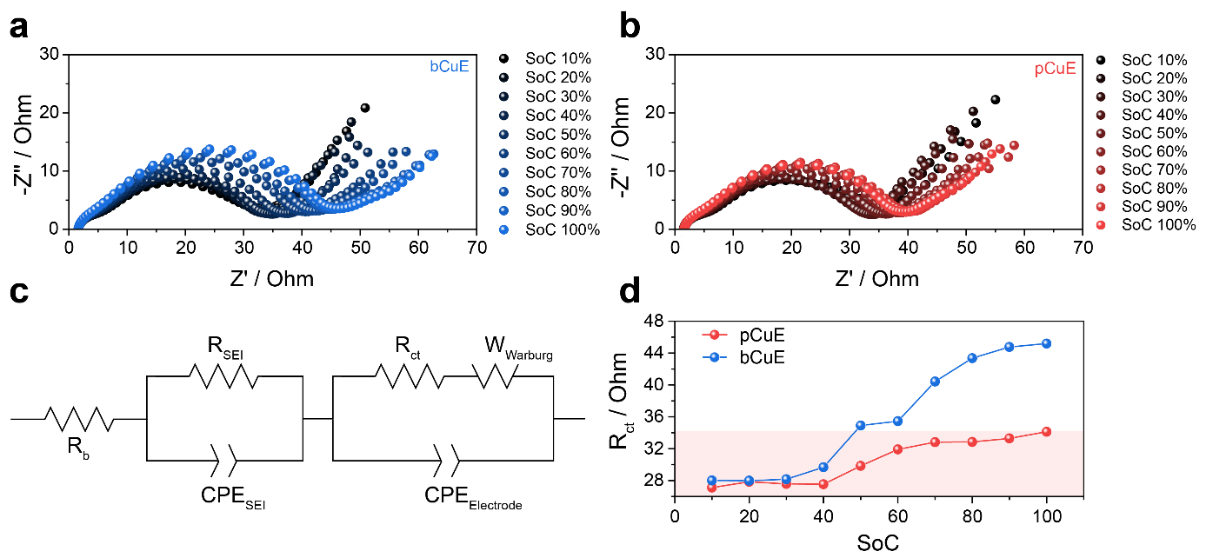


**Fig. S13** Nyquist plot of electrochemical impedance spectra measured from 10% to 100% SoC in 10% increments after pre-cycling of **a** the pCuE half-cell and **b** the bCuE half-cell. **c** Equivalent circuit model for characterizing charge transfer resistance from EIS spectra. **d** Corresponding charge transfer resistance (R_ct_) as a function of SoC


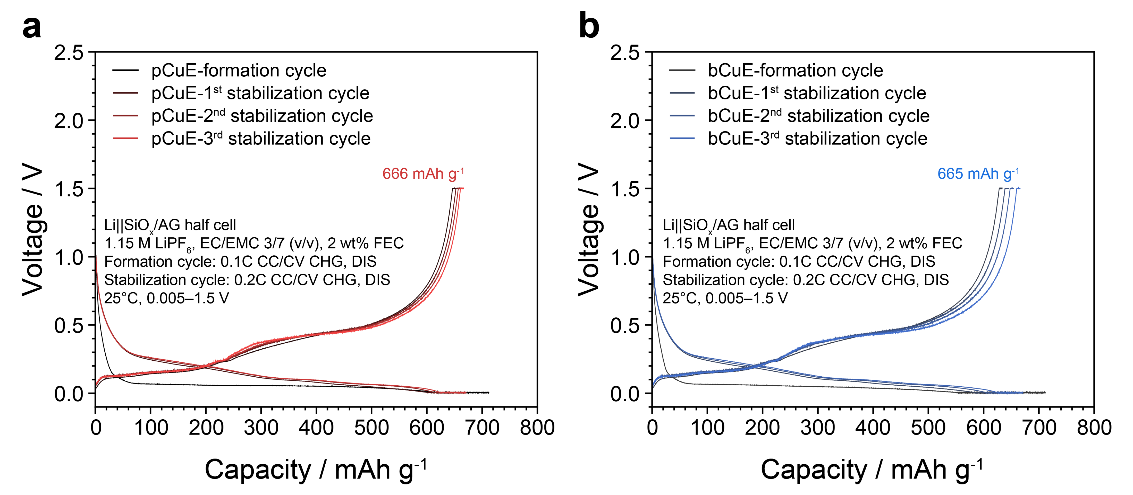


**Fig. S14** **a** Voltage profile of the pCuE and **b** voltage profile of the bCuE during precycling in the half-cell configuration


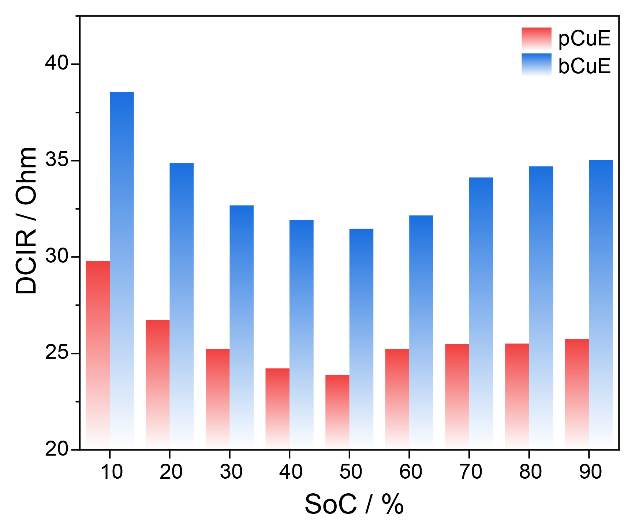


**Fig. S15** Direct current internal resistance (DCIR) measured from 10% to 100% SoC in 10% increments after pre-cycling using hybrid pulse power characterization (HPPC) protocol


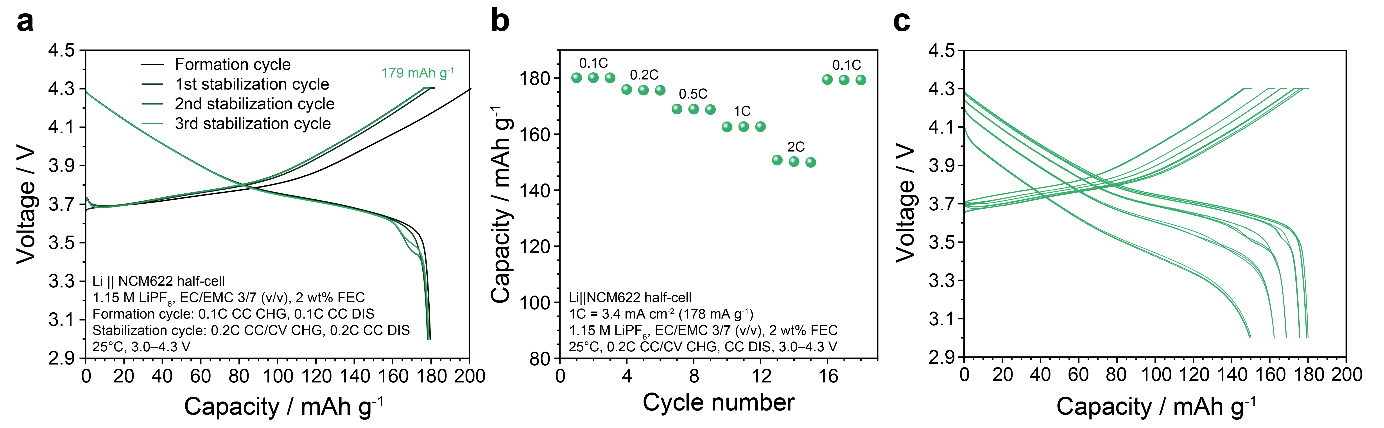


**Fig. S16 a** Voltage profile of the NCM622 half-cell during precycling. **b** Discharge capacities and **c** corresponding voltage profiles of NCM622 half-cells under various discharge rates (0.1C, 0.2C, 0.5C, 1C, and 2C).


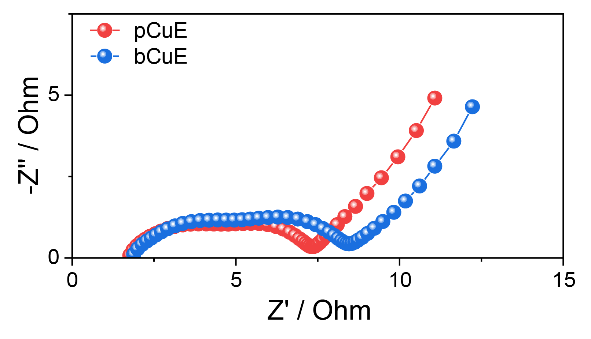


**Fig. S17** Nyquist plot of electrochemical impedance spectra measured at 50% SoC after pre-cycling of the pCuE and the bCuE full cells


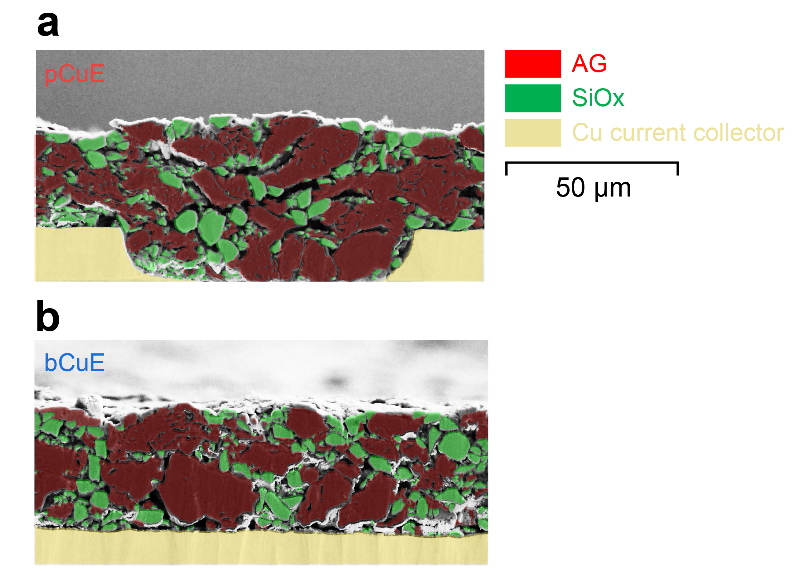


**Fig. S18** Segmentation results for each electrode component (AG, SiOx, and Cu current collector) of **a** the pCuE and **b** the bCuE for electrochemical modeling and simulation


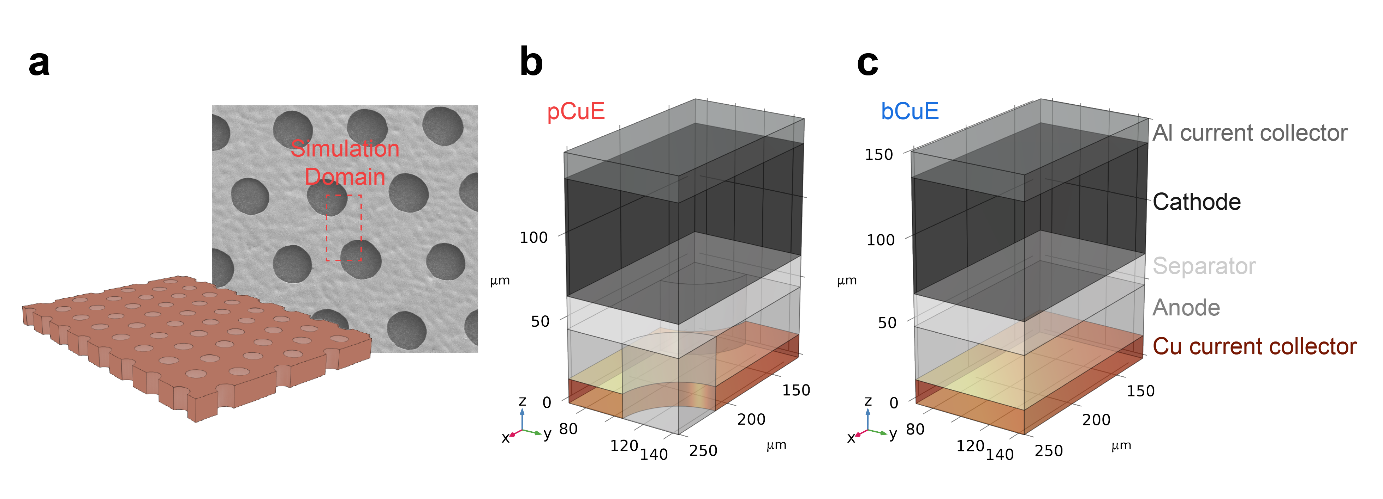


**Fig. S19 a** Pseudo-four-dimensional (P4D) electrochemical modeling and simulation domain and geometry of **b** the virtual pCuE and **c** bCuE full cells


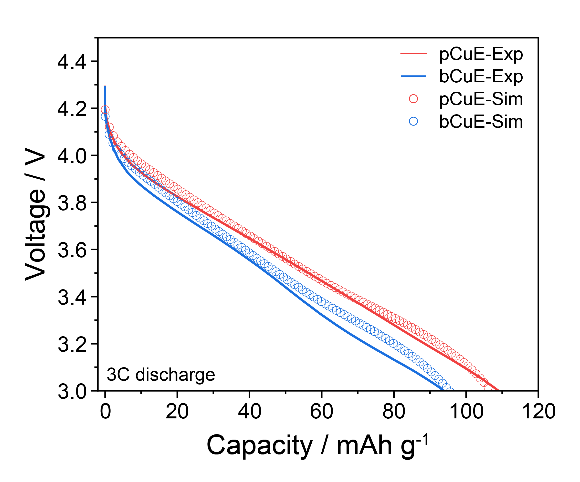


**Fig. S20** Discharge voltage profile obtained from experiment and simulation


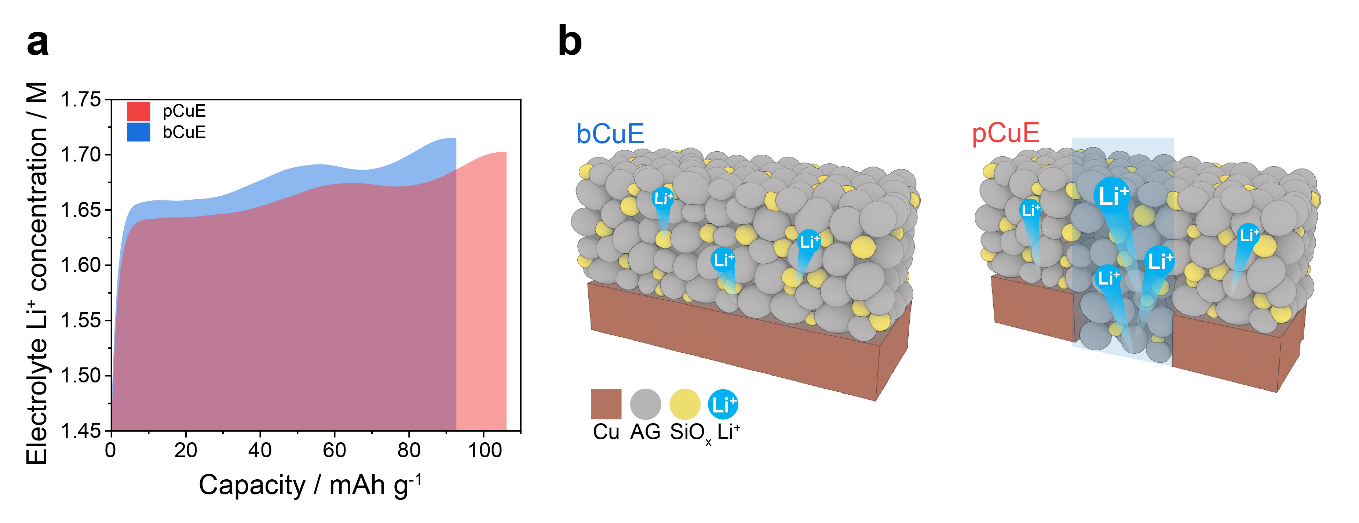


**Fig. S21 a** Lithium-ion concentration curve in the electrode domain as a function of discharge capacity. **b** Schematic illustration of lithium-ion transport characteristics in the bCuE and the pCuE


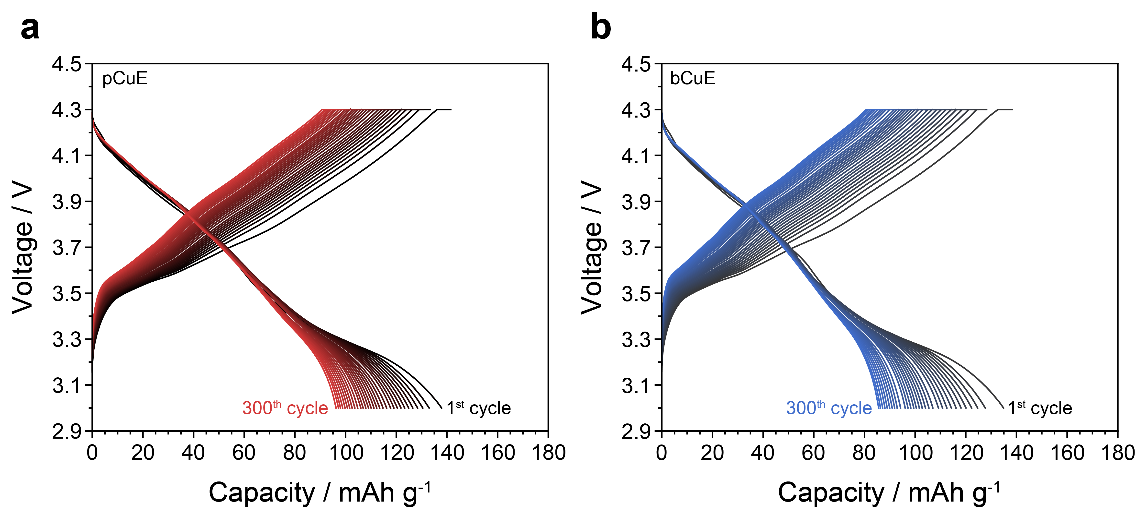


**Fig. S22** Voltage profiles of the **a** pCuE and **b** bCuE full cells during 0.5C cycling


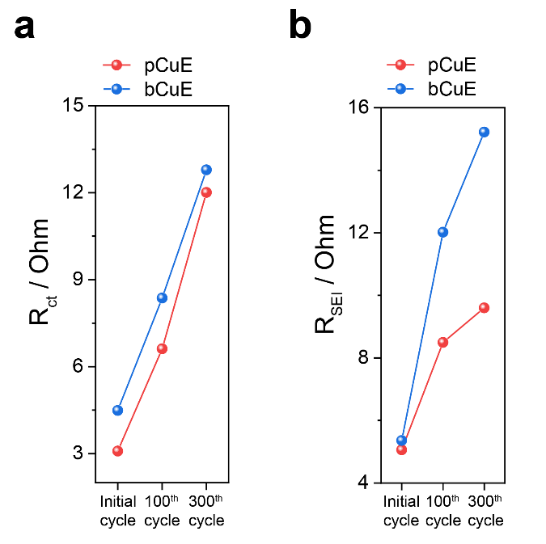


**Fig. S23 a** Charge transfer resistance and **b** SEI resistance estimated form the electrochemical impedance spectra measured before cycling, after 100 cycles, and after 300 cycles of the pCuE and the bCuE full cells at the discharge state using equivalent circuit model

**
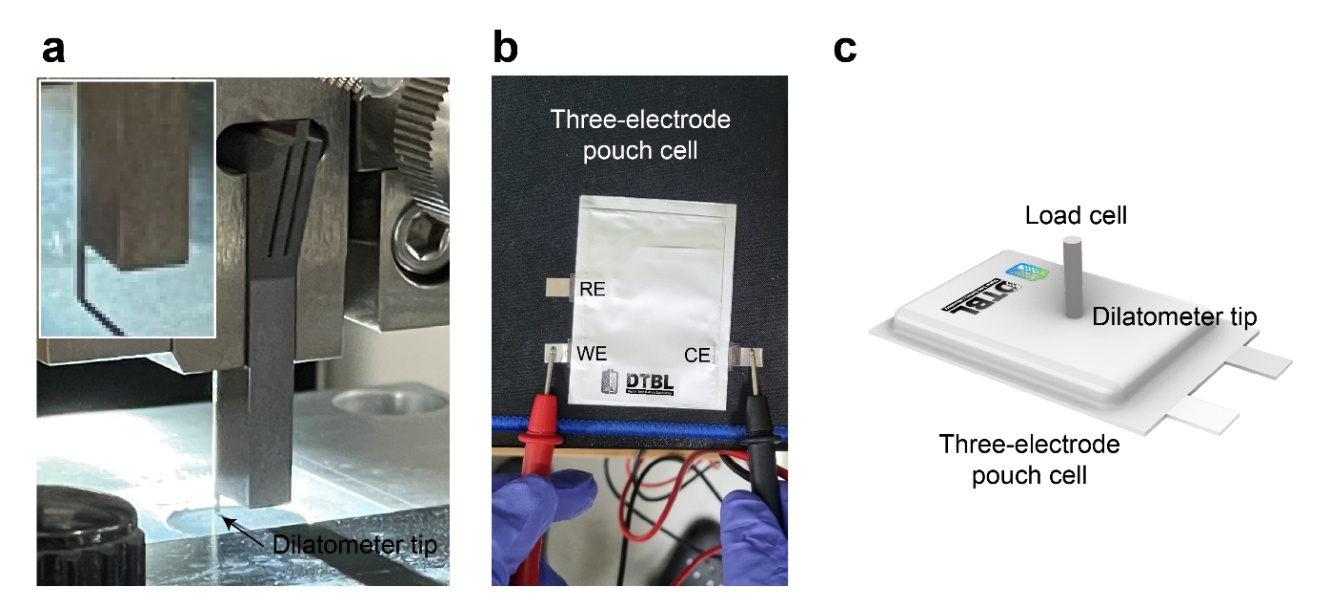
**

**Fig. S24** Optical image of **a** the dilatometry measurement setup and **b** the three-electrode pouch cell. **c** Schematic illustration of the dilatometry measurement


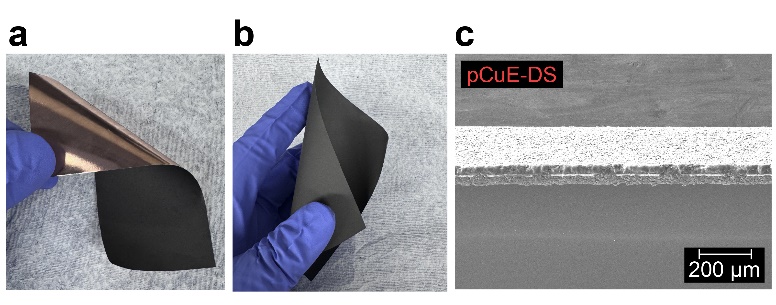


**Fig. S25** **a** Digital camera image of **a** the single-sided pCuE and **b** the double-sided pCuE (pCuE-DS). **c, d** Cross-sectional SEM images of the pCuE-DS


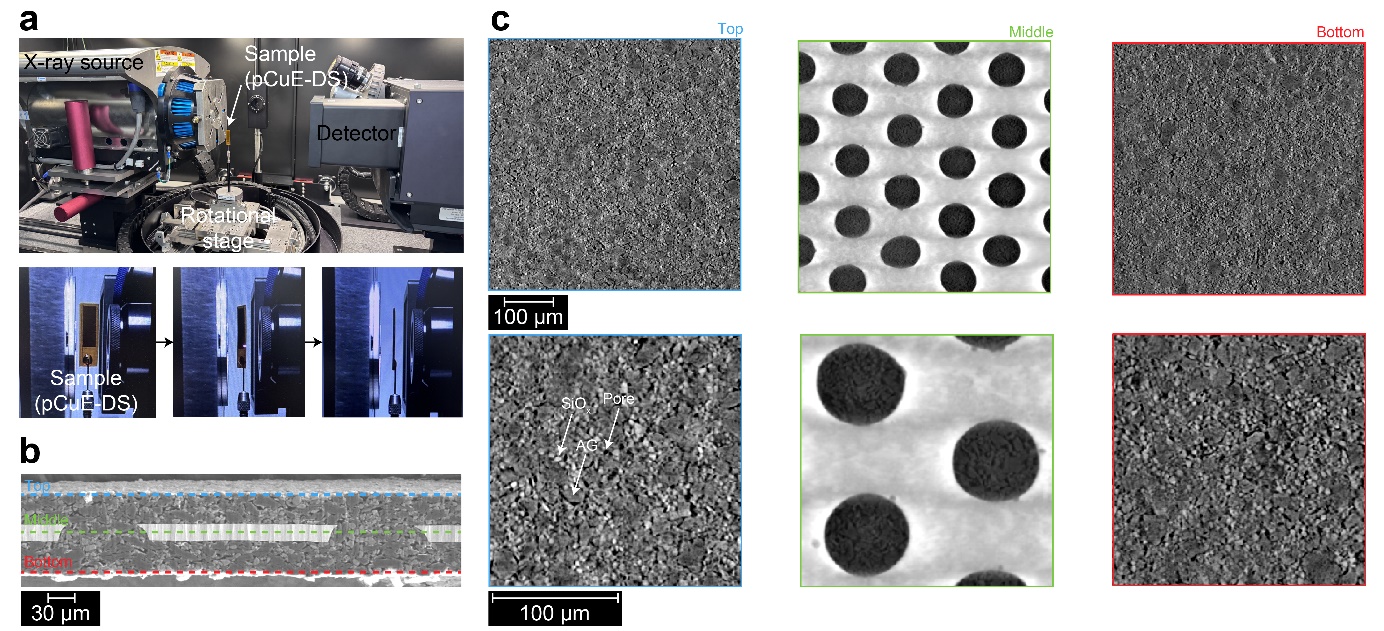


**Fig. S26** **a** Digital camera image of the XCT measurement setup. **b** Cross-sectional SEM image of the double-sided pCu electrode (pCuE-DS). **c** XCT tomographic images of the pCuE-DS at different electrode depths (top, middle, and bottom layers)


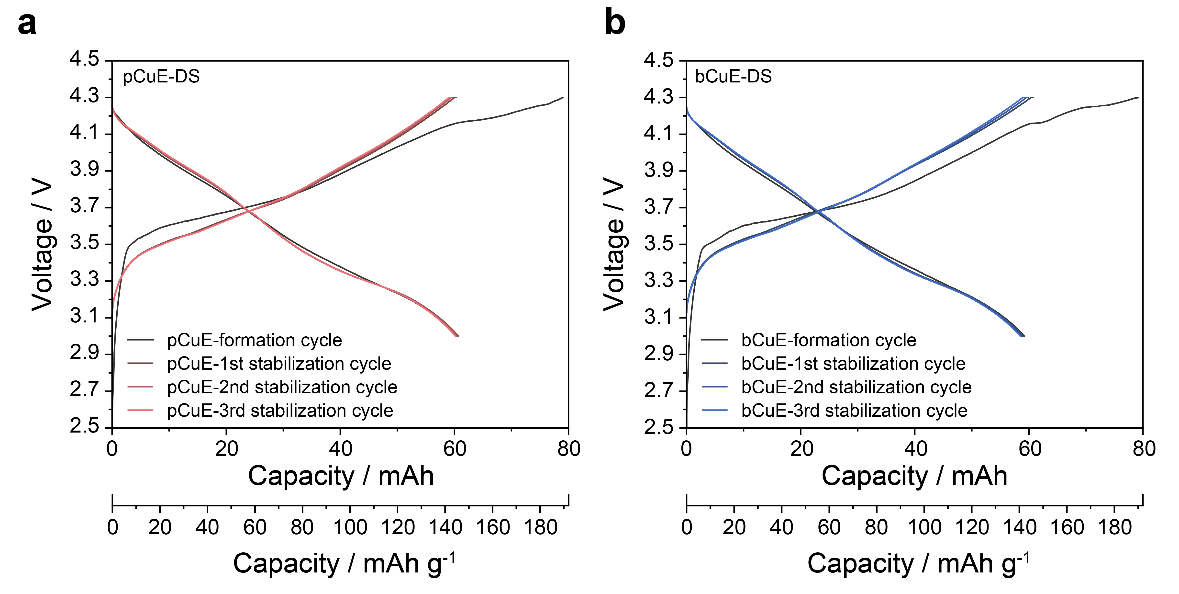


**Fig. S27** Voltage profile of **a** the pCuE-DS pouch-type full cell and **b** the bCuE-DS pouch-type full cell


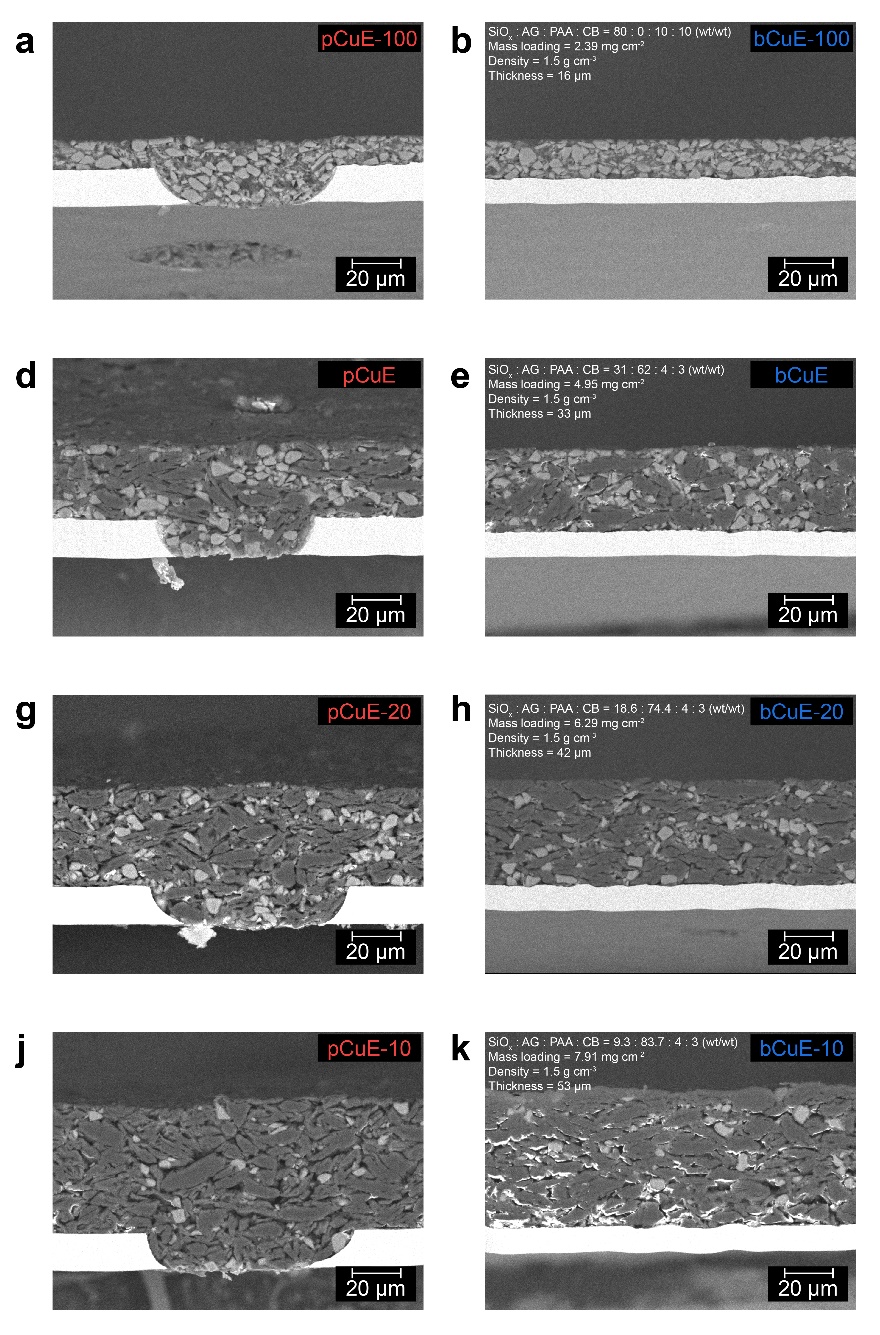


**Fig. S28** Cross-sectional SEM images of SiO_x_ electrodes composed of 100 wt% SiO_x_ as the active material with **a** pCu (pCuE-100) and **b** bCu (bCuE-100). SiO_x_/AG electrodes with 30 wt% SiO_x_ as the active material with **c** pCu (pCuE) and **d** bCu (bCuE). SiO_x_/AG electrodes with 20 wt% SiO_x_ as the active material with **g** pCu (pCuE-20) and **h** bCu (bCuE-20). SiO_x_/AG electrodes with 10 wt% SiO_x_ as the active material with **j** pCu (pCuE-10) and **k** bCu (bCuE-10)


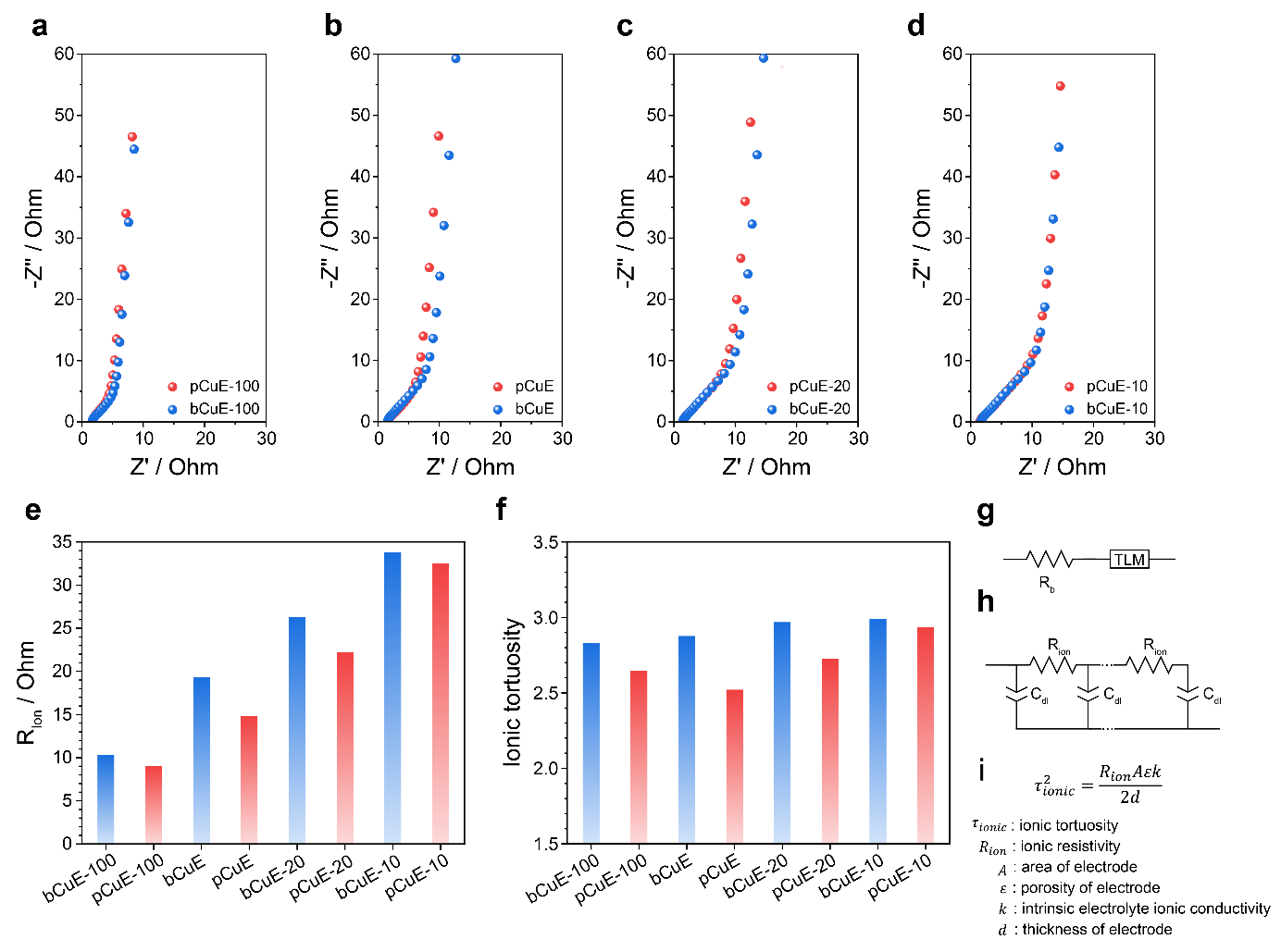


**Fig. S29** Symmetric cell EIS spectra of **a** pCuE-100 and bCuE-100, **b** pCuE and bCuE, **c** pCuE-20 and bCuE-20, and **d** pCuE-10 and bCuE-10. **e** Ionic resistance and **f** ionic tortuosity values calculated from the symmetric cell EIS spectra. **g** Equivalent circuit model and **h** transmission line model (TLM) used for estimating R_ion_. **i** Equation used to calculate ionic tortuosity based on Rᵢₒₙ [S1, S2]


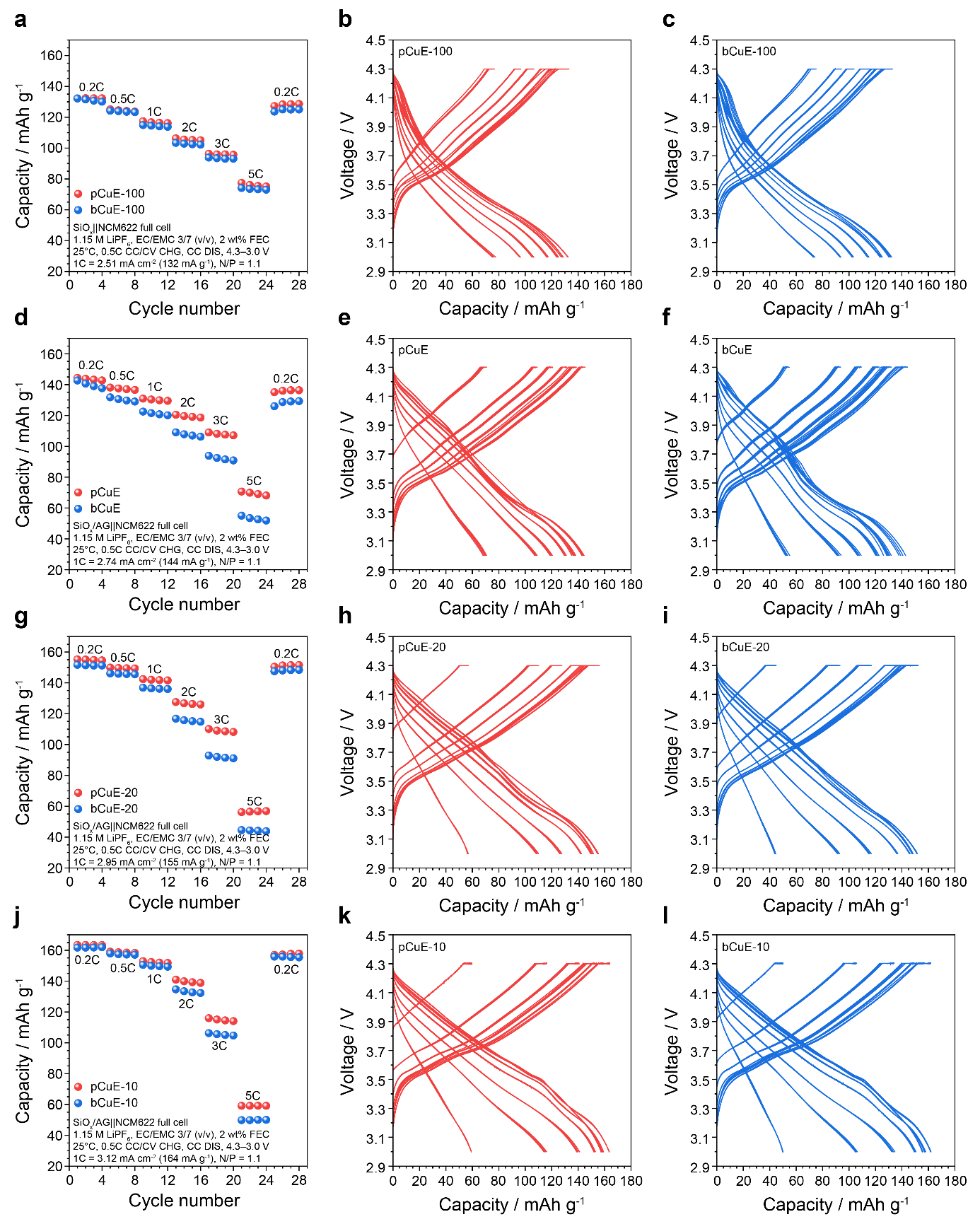


**Fig. S30** Rate performance of full cells with varying SiO_x_ content. **a–c** pCuE-100 and bCuE-100: discharge capacity (**a**) and voltage profiles (**b**, **c**). **d–f** pCuE and bCuE: discharge capacity (**d**) and voltage profiles (**e**, **f**). **g–i** pCuE-20 and bCuE-20: discharge capacity (**g**) and voltage profiles (**h**, **i**). **j–l** pCuE-10 and bCuE-10: discharge capacity (**j**) and voltage profiles (**k**, **l**)

**Supplementray Note S1**

we quantitatively estimated the gravimetric and volumetric energy densities of full cells assembled with pCuE and bCuE, as shown in Figs. 6f and 6g. The calculations were based on a single-side coated pouch-type cell configuration, assuming the following conditions:

- 10 µm-thick Cu current collector (pCu or bCu),

- 15 µm-thick Al current collector (Sam-A, Republic of Korea),

- 20 µm-thick separator (F20BHE, Tonen, Japan),

- cathode areal capacity: 3.3 mAh cm^-2^,

- N/P ratio: 1.1,

- initial Coulombic efficiency: 75%,

- nominal cell voltage: 3.75 V.

The compositions and densities of the cathode and anode were taken directly from those used in this study. The pouch and lead tabs were excluded from the energy density calculations to focus purely on internal components.


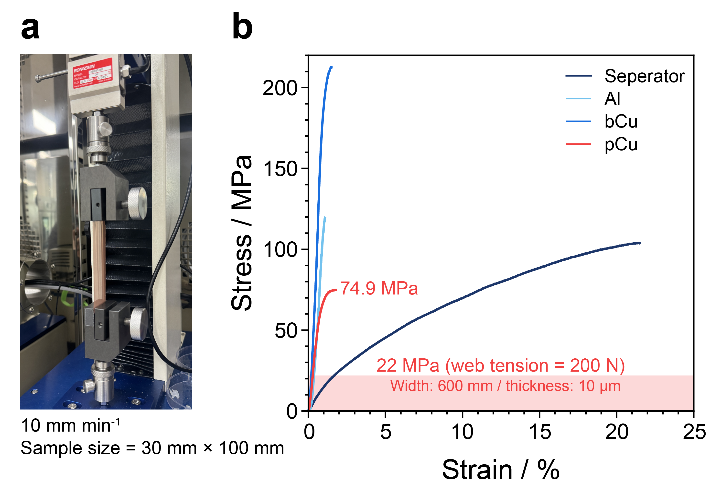


**Fig. S31** **a** Digital photograph of the experimental setup used for the tensile test of pCu. **b** Stress–strain curves of pCu, bCu, aluminum foil, and separator obtained from tensile test

**Table S1** Electrode design parameters for SiO­_x_/AG anodes

| Material | SiOx | AG | PAA | CMC | Super P | |
| --- | --- | --- | --- | --- | --- | --- |
| Composition / wt% | 31 | 62 | 3 | 0.5 | 3 | |
| Loading level / mg cm^-2^ | 4.95 | | | | | |
| Thickness / µm | 33/30^*^ | | | | | |
| Density / g cm^-3^ | 1.5 | | | | | |
| Areal capacity / mAh cm^-2^ | 3.6 | | | | | |
| ^* pCuE, thickness above the current collector surface^ | | | | | |  |

**Table S2** Electrode design parameters for NCM622 cathode

| Material | NCM622 | PVDF | Super P | |
| --- | --- | --- | --- | --- |
| Composition / wt% | 96 | 2 | 2 | |
| Loading level / mg cm^-2^ | 19.8 | | | |
| Thickness / µm | 69 | | | |
| Density / g cm^-3^ | 3.5 | | | |
| Areal capacity / mAh cm^-2^ | 3.3^*^ | | | |
| ^* N/P ratio: ~1.1^ | | | |  |

**Table S3** Governing equations of pseudo-four-dimensional electrochemical model

| **Electrochemical reaction** | **Governing equation** | **Domain** |
| --- | --- | --- |
| Diffusion and migration | $\frac{\partial c_{e}}{\partial t}=\nabla\cdot\left( D_{e}^{\mathrm{eff}}\nabla c_{e} \right)-\nabla\cdot\frac{t_{+}\vec{j}_{e}}{F}$ | Electrolyte |
| Charge conservation  and electroneutrality | $\nabla\cdot\vec{j}_{e}=\nabla\cdot\left[ \sigma_{e}^{\mathrm{eff}}(1-t_{+})\frac{2RT}{F}\left( 1+\frac{\partial lnf}{\partial lnc_{e}} \right)\nabla lnc_{e} \right]$  $-\nabla\cdot(\sigma_{e}^{\mathrm{eff}}\nabla\phi_{e})$ | Electrolyte |
| Diffusion | $\frac{\partial c_{s}}{\partial t}=D_{s}^{\mathrm{eff}}\left( \frac{\partial^{2}c_{s}}{\partial r^{2}}+\frac{2}{r}\frac{\partial c_{s}}{\partial r} \right)$ | Active material |
| Ohm’s law | $\nabla\cdot\vec{j}_{s}=-\nabla\cdot(\sigma_{s}^{\mathrm{eff}}\nabla\phi_{s})$ |  |
| Butler-Volmer  equation | $i_{\mathrm{se}}=2i_{0}\sinh\left[ (\phi_{s}-\phi_{e}-E_{\mathrm{eq}})\frac{F}{2RT} \right]$  $\vec{j}_{e}\vec{n}={a_{s}i}_{\mathrm{se}}$  $\vec{j}_{s}\vec{n}={a_{s}i}_{\mathrm{se}}$ | Electrode/  Electrolyte  interface |

**Table S4** Initial and boundary condition of pseudo-four-dimensional electrochemical model

| **Initial and boundary condition** | |  |
| --- | --- | --- |
| Electrolyte mass transport | ${c_{e}\vert}_{t=0}=c_{e,ini}$  ${{\nabla c}_{e}\vert}_{x=0}={{\nabla c}_{e}\vert}_{x=L_{x}}=0$  ${{\nabla c}_{e}\vert}_{y=0}={{\nabla c}_{e}\vert}_{y=L_{y}}=0$  ${{\nabla c}_{e}\vert}_{z=0}={{\nabla c}_{e}\vert}_{z=L_{z}}=0$  ${{{-D}_{e}^{\mathrm{eff}}\nabla c}_{e}\vert}_{x=L_{a}}={-D}_{e}^{\mathrm{eff}}{{\nabla c}_{e}\vert}_{x=L_{a}+L_{\mathrm{sep}}}$ | |
| Solid mass transport | ${c_{s}\vert}_{t=0}=c_{s,ini}$  $\left. \frac{\partial c_{s}}{\partial r} \right\vert_{r=0}=0$  $D_{s}^{\mathrm{eff}}\left. \frac{\partial c_{s}}{\partial r} \right\vert_{r=r_{s}}=\frac{{a_{s}i}_{\mathrm{se}}}{F}$ | |
| Electrolyte electric filed | ${{\nabla\phi}_{e}\vert}_{x=0}={{\nabla\phi}_{e}\vert}_{x=L_{x}}=0$  ${{\nabla\phi}_{e}\vert}_{y=0}={{\nabla\phi}_{e}\vert}_{y=L_{y}}=0$  ${{\nabla\phi}_{e}\vert}_{z=0}={{\nabla\phi}_{e}\vert}_{z=L_{z}}=0$  $\left( \sigma_{e}^{eff}\nabla^{2}\phi_{e}+FD_{e}^{eff}\nabla^{2}c_{e} \right)\vert_{x=L_{a}}=\left( \sigma_{e}^{eff}\nabla^{2}\phi_{e}+FD_{e}^{eff}\nabla^{2}c_{e} \right)\vert_{x=L_{a}+L_{sep}}$ | |
| Solid electric field | ${{\nabla\phi}_{s}\vert}_{x=0}={{\nabla\phi}_{s}\vert}_{x=L_{x}}=0$  ${{\nabla\phi}_{s}\vert}_{y=0}={{\nabla\phi}_{s}\vert}_{y=L_{y}}=0$  ${{\nabla\phi}_{s}\vert}_{z=0}={{\nabla\phi}_{s}\vert}_{z=L_{z}}=0$  ${{\nabla\phi}_{s}\vert}_{x=L_{a}}={{\nabla\phi}_{s}\vert}_{x=L_{a}+L_{\mathrm{sep}}}$  $-\sigma_{s}^{\mathrm{eff}}\nabla\phi_{s}\vert_{x=0}=\sigma_{s}^{\mathrm{eff}}\nabla\phi_{s}\vert_{x=L_{x}}=\vec{j}_{s}$  $\eta=\phi_{s}-\phi_{e}-E_{\mathrm{eq}}$ | |

**Table S5** Model parameters

| Symbol | Value | | Unit |
| --- | --- | --- | --- |
| $c_{e,ini}$ | 1.15 | | M (mol L^−1^) |
| $c_{max}$ | NCM622 | 24,290^[a]^ | mol m^−3^ |
|  | SiO_x_/AG | 57,490 ^[a]^ |  |
| $E_{eq}$ | NCM622 | [3] | V |
|  | SiO_x_/AG | [4] |  |
| $\sigma_{e}$ | 9.3275[5] | | mS cm^−1^ |
| $\sigma_{s}$ | Cathode | 0.017^[b]^ | S cm^−1^ |
|  | Anode | 18.87^[b]^ |  |
| $D_{e}$ | 3.8346×10^−10^[5] | | m^2^ s^−1^ |
| $D_{s}$ | NCM622 | 2×10^−13^[3] | m^2^ s^−1^ |
|  | SiO_x_/AG | 2.619×10^−14^[6] |  |
| $t_{+}$ | 0.250[7] | | - |
| $i_{0}$ | NCM622 | 0.35^[c]^ | mA cm^−2^ |
|  | SiO_x_/AG | 0.48^[c]^ |  |
| $T$ | 298.15^[b]^ | | K |
| $R$ | 8.314 | | J mol^−1^ K^−1^ |
| $F$ | 96485 | | C mol^−1^ |

*[a] Theoretically calculated*

*[b] Experimentally measured*

*[c] Fitting parameter*

**Table S6** Glossary of symbols

| **Symbol** | **Description** | **Unit** |
| --- | --- | --- |
| $c_{e}$ | Lithium-ion concentration in electrolyte | mol m^-3^ |
| $c_{s}$ | Lithium-ion concentration in active material | mol m^-3^ |
| $c_{\max}$ | Maximum lithium-ion concentration in active material | mol m^-3^ |
| $J_{e}$ | Current density in electrolyte | A cm^-2^ |
| $J_{s}$ | Current density in active material | A cm^-2^ |
| $i_{\mathrm{se}}$ | Butler-Volmer interface current density | A cm^-2^ |
| $\phi_{e}$ | Electric potential of electrolyte | V |
| $\phi_{s}$ | Electric potential of active material | V |
| $E_{\mathrm{eq}}$ | Equilibrium potential of active material (vs Li/Li+) | V |
| $\sigma_{e}$ | Ionic conductivity of electrolyte | S m^-1^ |
| $\sigma_{s}$ | Electronic conductivity of active material | S m^-1^ |
| $D_{e}$ | Lithium-ion diffusion coefficient of electrolyte | m^2^ s^-1^ |
| $D_{s}$ | Lithium-ion diffusion coefficient of active material | m^2^ s^-1^ |
| $t_{+}$ | Lithium-ion transference number of electrolyte | - |
| $i_{0}$ | Exchange current density | A cm^-2^ |
| $\eta$ | Overpotential | V |
| $L_{a}$ | Anode thickness | µm |
| $L_{\mathrm{sep}}$ | Separator thickness | µm |
| $L_{c}$ | Cathode thickness | µm |
| $T$ | Temperature | K |
| $R$ | Universal gas constant (8.3143) | J mol^-1^ K^-1^ |
| $F$ | Faraday constant (96,487) | C mol^-1^ |
| $\mathrm{ini}$ | Initial state | - |
| $\mathrm{eff}$ | Effective parameter | - |

**Table S7.** Electrode design parameters and cycling performance of SiOₓ-based composite anodes reported in previous literature.

| Cathode | | Anode | | | |  |  |  |  |
| --- | --- | --- | --- | --- | --- | --- | --- | --- | --- |
| Active  material | **Loading level**  **(mg cm^-2^)** | **Active**  **material** | **Capacity (mAh g^-1^)** | **Loading level**  **(mg cm^-2^)** | **Density**  **(g cm^-3^)** | **NP ratio** | **Cut-off voltage** | **Capacity retention** | **Refs.** |
| LiNi_0.6_Co_0.2_Mn_0.2_O_2_ | **19.8** | **SiO_x_/AG** | **665** | **4.95** | **1.5** | **1.1** | **4.3** | **80%@**  **160 cyc** | **This work** |
| LiNi_0.6_Co_0.2_Mn_0.2_O_2_ | 14.1 | SiO_x_/Gr/C | 653 | 3.7 | 1.4 | 1.15 | 4.3 | 86%@  100 cyc | [S8] |
| LiNi_0.3_Co_0.3_Mn_0.3_O_2_ | N/A | SiO_x_ | 1252 | 3.3 | 1.0 | 1.1 | 4.3 | 62%@  100 cyc | [S9] |
| LiNi_0.8_Co_0.15_Al_0.05_O_2_ | 27.8 | SiO_x_/C | 1000 | 2.3 | 1.0 | 1.1 | 4.2 | 78%@  50 cyc | [S10] |
| LiNi_0.6_Co_0.2_Mn_0.2_O_2_ | 20 | SiO_x_@graphene  /Gr | 680 | 5.0 | 1.5 | 1.1 | 4.3 | 80%@  120 cyc | [S11] |
| LiNi_0.8_Co_0.1_Mn_0.1_O_2_ | 18.5 | SiO_x_/C@Gr | 650 | 7.0 | N/A | 1.1 | 4.2 | 93%@  100 cyc | [S12] |
| LiNi_0.8_Co_0.15_Al_0.05_O_2_ | 14 | SiO_x_/C | 978 | 2.2 | 0.9 | 1.1 | 4.2 | 61%@  100 cyc | [S13] |
| LiNi_0.5_Co_0.2_Al_0.3_O_2_ | 11.5 | SiO_x_/PPy | 1280 | 2.3 | N/A | N/A | 4.3 | 76%@  90 cyc | [S14] |
| LiNi_0.6_Co_0.2_Mn_0.2_O_2_ | 7.2 | SiO/C | 1500 | 0.9 | N/A | 1.2 | 4.2 | 49%@  40 cyc | [S15] |
| LiNi_0.8_Co_0.1_Mn_0.1_O_2_ | 6.0 | Si@SiO_2_@C | 972 | 1.0 | 0.32 | 1.2 | 4.2 | 61%@  100 cyc | [S16] |
| LiFePO_4_ | 6.0 | SiO/C | 1500 | 0.9 | N/A | 1.4 | 4.2 | 52%@  100 cyc | [S15] |
| LiCoO_2_ | 5.5 | SiO_x_ | 1145 | 0.8 | N/A | 1.1 | 4.3 | 90%@  50 cyc | [S17] |
| LiCoO_2_ | 4.0 | Si/SiO_2_ | 873 | 0.7 | N/A | 1.2 | 4.0 | 60%@  100 cyc | [S18] |
| LiFePO_4_ | N/A | SiO_x_/C | 1568 | N/A | N/A | 1.15 | 3.8 | 94%@  100 cyc | [S19] |

**Supplementary References**

1. N. Ogihara, Y. Itou, T. Sasaki, Y. Takeuchi, Impedance spectroscopy characterization of porous electrodes under different electrode thickness using a symmetric cell for high-performance lithium-ion batteries. J. Phys. Chem. C **119**(9), 4612–4619 (2015). <https://doi.org/10.1021/jp512564f>
2. A. Shodiev, M. Chouchane, M. Gaberscek, O. Arcelus, J. Xu et al., Deconvoluting the benefits of porosity distribution in layered electrodes on the electrochemical performance of Li-ion batteries. Energy Storage Mater. **47**, 462–471 (2022). <https://doi.org/10.1016/j.ensm.2022.01.058>
3. O. Chaouachi, J.-M. Réty, S. Génies, M. Chandesris, Y. Bultel, Experimental and theoretical investigation of Li-ion battery active materials properties: Application to a graphite/Ni_0.6_Mn_0.2_Co_0.2_O_2_ system. Electrochim. Acta **366**, 137428 (2021). <https://doi.org/10.1016/j.electacta.2020.137428>
4. D.K. Karthikeyan, G. Sikha, R.E. White, Thermodynamic model development for lithium intercalation electrodes. J. Power Sources **185**(2), 1398–1407 (2008). <https://doi.org/10.1016/j.jpowsour.2008.07.077>
5. T.G. Zavalis, M. Behm, G. Lindbergh, Investigation of short-circuit scenarios in a lithium-ion battery cell. J. Electrochem. Soc. **159**(6), A848–A859 (2012). <https://doi.org/10.1149/2.096206jes>
6. K. Kumaresan, G. Sikha, R.E. White, Thermal model for a Li-ion cell. J. Electrochem. Soc. **155**(2), A164 (2008). <https://doi.org/10.1149/1.2817888>
7. A. Nyman, M. Behm, G. Lindbergh, Electrochemical characterisation and modelling of the mass transport phenomena in LiPF_6_–EC–EMC electrolyte. Electrochim. Acta **53**(22), 6356–6365 (2008). <https://doi.org/10.1016/j.electacta.2008.04.023>
8. G. Li, J.-Y. Li, F.-S. Yue, Q. Xu, T.-T. Zuo et al., Reducing the volume deformation of high capacity SiO_x_/G/C anode toward industrial application in high energy density lithium-ion batteries. Nano Energy **60**, 485–492 (2019). <https://doi.org/10.1016/j.nanoen.2019.03.077>
9. K. Zhang, W. Du, Z. Qian, L. Lin, X. Gu et al., SiO_x_ embedded in N-doped carbon nanoslices: a scalable synthesis of high-performance anode material for lithium-ion batteries. Carbon **178**, 202–210 (2021). <https://doi.org/10.1016/j.carbon.2021.03.011>
10. Y. Cho, J. Kim, A. Elabd, S. Choi, K. Park et al., A pyrene-poly(acrylic acid)-polyrotaxane supramolecular binder network for high-performance silicon negative electrodes. Adv. Mater. **31**(51), e1905048 (2019). <https://doi.org/10.1002/adma.201905048>
11. L. Lee, W. To A Ran, J.-H. Lee, S.M. Hwang, Y.-J. Kim, Self-adaptive anode design with graphene-coated SiOx/graphite for high-energy Li-ion batteries. Chem. Eng. J. **442**, 136166 (2022). <https://doi.org/10.1016/j.cej.2022.136166>
12. M.-Y. Yan, G. Li, J. Zhang, Y.-F. Tian, Y.-X. Yin et al., Enabling SiOx/C anode with high initial coulombic efficiency through a chemical pre-lithiation strategy for high-energy-density lithium-ion batteries. ACS Appl. Mater. Interfaces **12**(24), 27202–27209 (2020). <https://doi.org/10.1021/acsami.0c05153>
13. H.J. Kim, S. Choi, S.J. Lee, M.W. Seo, J.G. Lee et al., Controlled prelithiation of silicon monoxide for high performance lithium-ion rechargeable full cells. Nano Lett. **16**(1), 282–288 (2016). <https://doi.org/10.1021/acs.nanolett.5b03776>
14. S. Fang, N. Li, T. Zheng, Y. Fu, X. Song et al., Highly graphitized carbon coating on SiO with a π⁻π stacking precursor polymer for high performance lithium-ion batteries. Polymers **10**(6), 610 (2018). <https://doi.org/10.3390/polym10060610>
15. B. Huang, T. Huang, L. Wan, A. Yu, Pre-lithiating SiO anodes for lithium-ion batteries by a simple, effective, and controllable strategy using stabilized lithium metal powder. ACS Sustainable Chem. Eng. **9**(2), 648–657 (2021). <https://doi.org/10.1021/acssuschemeng.0c05851>
16. Y. Zhang, B. Li, B. Tang, Z. Yao, X. Zhang et al., Mechanical constraining double-shell protected Si-based anode material for lithium-ion batteries with long-term cycling stability. J. Alloys Compd. **846**, 156437 (2020). <https://doi.org/10.1016/j.jallcom.2020.156437>
17. H. Wu, L. Zheng, J. Zhan, N. Du, W. Liu et al., Recycling silicon-based industrial waste as sustainable sources of Si/SiO_2_ composites for high-performance Li-ion battery anodes. J. Power Sources **449**, 227513 (2020). <https://doi.org/10.1016/j.jpowsour.2019.227513>
18. J. Liang, X. Li, Z. Hou, W. Zhang, Y. Zhu et al., A deep reduction and partial oxidation strategy for fabrication of mesoporous Si anode for lithium ion batteries. ACS Nano **10**(2), 2295–2304 (2016). <https://doi.org/10.1021/acsnano.5b06995>
19. Q. Sun, J. Li, C. Hao, L. Ci, Focusing on the subsequent coulombic efficiencies of SiO*_x_*: initial high-temperature charge after over-capacity prelithiation for high-efficiency SiO*_x_*-based full-cell battery. ACS Appl. Mater. Interfaces **14**(12), 14284–14292 (2022). <https://doi.org/10.1021/acsami.2c01392>
